# Mixed Signal Learning by Spike Correlation Propagation in Feedback Inhibitory Circuits

**DOI:** 10.1371/journal.pcbi.1004227

**Published:** 2015-04-24

**Authors:** Naoki Hiratani, Tomoki Fukai

**Affiliations:** 1 Department of Complexity Science and Engineering, The University of Tokyo, Kashiwa, Chiba, Japan; 2 Laboratory for Neural Circuit Theory, RIKEN Brain Science Institute, Wako, Saitama, Japan; Research Center Jülich, GERMANY

## Abstract

The brain can learn and detect mixed input signals masked by various types of noise, and spike-timing-dependent plasticity (STDP) is the candidate synaptic level mechanism. Because sensory inputs typically have spike correlation, and local circuits have dense feedback connections, input spikes cause the propagation of spike correlation in lateral circuits; however, it is largely unknown how this secondary correlation generated by lateral circuits influences learning processes through STDP, or whether it is beneficial to achieve efficient spike-based learning from uncertain stimuli. To explore the answers to these questions, we construct models of feedforward networks with lateral inhibitory circuits and study how propagated correlation influences STDP learning, and what kind of learning algorithm such circuits achieve. We derive analytical conditions at which neurons detect minor signals with STDP, and show that depending on the origin of the noise, different correlation timescales are useful for learning. In particular, we show that non-precise spike correlation is beneficial for learning in the presence of cross-talk noise. We also show that by considering excitatory and inhibitory STDP at lateral connections, the circuit can acquire a lateral structure optimal for signal detection. In addition, we demonstrate that the model performs blind source separation in a manner similar to the sequential sampling approximation of the Bayesian independent component analysis algorithm. Our results provide a basic understanding of STDP learning in feedback circuits by integrating analyses from both dynamical systems and information theory.

## Author Summary

In natural environments, although sensory inputs are often highly mixed with one another and obscured by noise, animals can detect and learn discrete signals from this mixture. For example, humans easily detect the mention of their names from across a noisy room, a phenomenon known as the cocktail party effect. Spike-timing-dependent plasticity (STDP) is a learning mechanism ubiquitously observed in the brain across various species and is considered to be the neural basis of such learning; however, it is still unclear how STDP enables efficient learning from uncertain stimuli and whether spike-based learning offers benefits beyond those provided by standard machine learning methods for signal decomposition. To begin to answer these questions, we conducted analytical and simulation studies examining the propagation of spike correlation in feedback neural circuits. We show that non-precise spike correlation is useful for handling noise during the learning process. Our results also suggest that neural circuits make use of stochastic membrane dynamics to approximate computationally complex Bayesian learning algorithms, progressing our understanding of the principles of stochastic computation by the brain.

## Introduction

Neurons receive inputs from a large number of other neurons encoding a variety of information about various signals. Despite the diversity and variability of input spike trains, neurons can learn and represent specific information during developmental processes and according to specific task requirements. Spike-timing-dependent plasticity (STDP) [1,2] is a candidate mechanism of neural learning. Extensive studies have revealed the type of information that a single neuron can learn through STDP [3–7]; however, the type of information that a population of neurons interacting with each other learns through STDP has not yet been determined. Understanding this extension from a single neuron to a population of neurons is crucial because a single neuron learns and represents only a limited amount of information that may be transmitted to it from thousands of inputs.

Among neural interactions, lateral inhibition is a basic interaction widely observed in various regions, such as the olfactory bulb [8], visual cortex [9], somatosensory cortex [10], and entorhinal cortex [11]. Previous theoretical results showed that neural circuits with lateral inhibition enhance signal detection [12,13] and improve learning performance [14–16]. Several simulation studies further revealed that neurons acquire receptive field [17–19] or spike patterns [20] through STDP by introducing lateral inhibition; yet, those studies were limited to simplified cases for which a large population of independent neurons was suggested to be sufficient [5,21,22]. Therefore, it remains unclear whether lateral inhibition plays a crucial role in STDP learning; in particular, the spike level effects of lateral inhibition remain elusive. Moreover, recent experimental results suggest that animals learn and discriminate mixed olfactory signals [23–25] or auditory signals masked by noise [26,27], but it is still unknown how feedback interactions contribute to such learning.

Here, by considering a simple feedback network model of spiking neurons, we investigated the algorithm inherent to STDP in neural circuits containing feedback. We analyzed the propagation of spike correlations through inhibitory circuits, and revealed how such secondary correlations influence STDP learning at both feedforward and feedback connections. We discovered that the timescale of spike correlation preferable for learning depends on whether the noise is independent from any signal (random noise) or generated from the mixing of signals (cross-talk noise). We also found that excitatory and inhibitory STDP cooperatively shapes lateral circuit structure, making it suitable for signal detection. We further found a possible link between stochastic membrane dynamics and sampling process, which is necessary for neural approximation of learning algorithm of Bayesian independent component analysis (ICA). We applied our findings by demonstrating that STDP implements a spike-based solution in neural circuits for the cocktail party problem [26,28,29].

## Results

### Model

**Fig 1:**
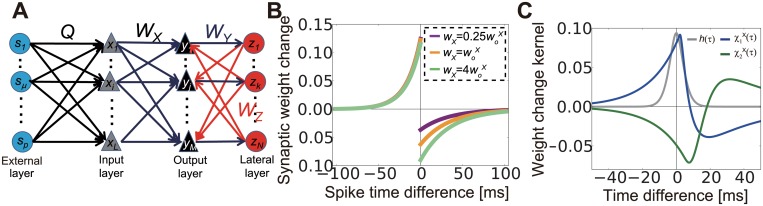
Description of the model. (A) Schematic figure of the model. (B) Spike-time dependent synaptic weight change in log- spike-timing-dependent plasticity (STDP). (C) Normalized temporal cross-correlogram of input neurons receiving common sources (gray line), and kernel functions of plasticity propagated by feedforward correlation (blue line) and feedback correlation (green line).

We constructed a network model with three feedforward layers as shown in Fig 1A (see Neural dynamics in Methods for details). The external source layer represents the external environment or neural activity at sensory systems. The external layer also provides common inputs to the input layer and induces correlations in the neurons in the input layer. The input layer shows rate-modulated Poisson firing based on events at the external layer and external noise, which is approximated with the constant firing rate {*r*_*i*_^*o*^}. Subsequently, spike activity at the input layer projects to the output layer, which also receives inhibitory feedback from the lateral layer. Neurons in the lateral layers are excited by inputs from the output layer. We assumed that all neurons in the input layer and the output layer are excitatory, whereas lateral-layer neurons are assumed to be inhibitory. Although excitatory lateral interactions also exist in the sensory cortex, they are typically sparse [30] and weak [10] compared with inhibitory interactions; thus we concentrated on the latter. For the analytical treatment, the neurons in the output and lateral layers were modeled with a linear Poisson model. We first studied synaptic plasticity at the feedforward connections (connections from the input layer to the output layer), while fixing lateral connections (i.e., connections from the output layer to the lateral layer and connections from the lateral layer to the output layer). For STDP, we used pairwise log-STDP (Fig 1B) [31], which replicates the experimentally observed long-tailed synaptic weight distribution [32,33].

We considered the case for information encoded in the correlated activity of input neurons [34,35], and fixed the average firing rate of all input neurons at the constant value *υ*_*o*_^*X*^ (See Table 1 and 2 for the list of variables and parameters). If the firing rate of input neuron *i* is given as $r_{i}^{o}+\sum_{\mu =1}^{p}q_{i\mu }\int_{0}^{\infty }\phi (t^{\prime })s_{\mu }(t-t^{\prime })dt^{\prime }$, for external event *s*_*μ*_ *(t)* and the response probability of the neuron *q*_*iμ*_, then common inputs from the external layer induce a temporal correlation proportional to

$$h(\tau ;\theta_{t})\equiv \int_{\max(\tau ,0)}^{\infty }dt^{\prime }\phi (t^{\prime })\phi (t^{\prime }-\tau ).$$ (1)

where φ*(t)* is a response kernel (see Eqs (14) and (24) in Methods for details). If we use $\phi (t)=t^{2}e^{-t/\theta_{t}}/2\theta_{t}{}^{3}$, where θ_t_ is the parameter that controls the timescale of spike correlations, then $h(\tau ;\theta_{t})=\frac{1}{16\theta_{t}{}^{3}}\left(\tau^{2}+3\theta_{t}\left|\tau \right|+3\theta_{t}{}^{2}\right)e^{-\left|\tau \right|/\theta_{t}}$ (gray line in Fig 1C). For the kernel function, we used the gamma distribution with shape parameter *k*_*g*_ = 3 in order to reproduce broad spike correlations typically observed in cortical neurons [36,37]. Synaptic weight dynamics by STDP is written as

$$\frac{dw_{ji}^{X}}{dt}=x_{i}(t-d_{ji}^{Xa})\int_{0}^{\infty }F_{d}(w_{ji}^{X},s)y_{j}(t-s-d_{ji}^{Xd})ds+y_{j}(t-d_{ji}^{Xd})\int_{0}^{\infty }F_{p}(w_{ji}^{X},s)x_{i}(t-s-d_{ji}^{Xa})ds$$

for $F_{d}(w_{ij}^{X},s)=f_{d}(w_{ij}^{X})e^{-s/\tau_{{}_{d}}}$, $F_{p}(w_{ij}^{X},s)=f_{p}(w_{ij}^{X})e^{-s/\tau_{{}_{p}}}$, where *f*_*d*_(*w*) and *f*_*p*_(*w*) are synaptic weight dependence of LTD/LTP (long-term depression/potentiation), respectively. By taking the average of above equation over time and ensemble (see Average synaptic weight velocity in Methods for details), the weight change of the feedforward connection *W*_*X*_ can be approximated as

$$\overset{•}{W_{X}}\approx W_{X}\left(g_{1}^{X}E-g_{2}^{X}W_{Z}W_{Y}\right)C^{t},$$ (2)

where g_1_^X^ and g_2_^X^ are scalar coefficients, *C* is the correlation matrix, and *E* is the identity matrix (see Eqs (25)–(30) for derivation). The first term describes the synaptic weight change directly caused by an input spike correlation and can be rewritten into the convolution of the temporal correlation and correlation kernel function χ^X^_1_ as

$$g_{1}^{X}\equiv G_{1}^{X}(w_{o}^{X}),G_{1}^{X}(w)\equiv \int_{-\infty }^{\infty }\chi_{1}^{X}(\tau ;w)h(\tau )d\tau ,\chi_{1}^{X}\left(\tau ;w\right)=\int_{-\tau +2d_{Xd}}^{\infty }dsF\left(w,s\right)\varepsilon_{X}\left(\tau +s-2d_{Xd}\right),$$ (3)

where *F*(*w*,*s*) = *F*_*d*_(*w*,*-s*) if *s*<0, else *F*(*w*,*s*) = *F*_*p*_(*w*,*s*), and *ε*_*X*_ is the EPSP curve of input neurons (see Eqs (15) and (31) in the Methods). By the deconvolution of *G*_1_^*X*^(*w*), we can separate the effect of the intrinsic network property χ^X^_1_ and that of the input correlation *h(τ)* for STDP-based learning. Due to causality, LTP/LTD balance, and dendritic delay, $\chi_{1}^{X}(\tau ;w)$ typically becomes LTP-dominant around τ = 0 (blue line in Fig 1C; we set *w* = *w*_*o*_^*X*^), so that *g*_1_^X^ takes positive values, which enables coincidence-based learning [4,5,38]. The second term of Eq (2), which is of particular interest in this model, describes how the input correlation influences STDP learning at feedforward connections through lateral inhibition:

$$g_{2}^{X}\equiv G_{2}^{X}(w_{o}^{X}),G_{2}^{X}(w)\equiv \int_{-\infty }^{\infty }\chi_{2}^{X}(\tau ;w)h(\tau )d\tau ,\chi_{2}^{X}\left(\tau ;w\right)=\int_{-\tau +D}^{\infty }dsF\left(w,s\right)\int_{0}^{\tau +s-D}dr\varepsilon_{Z}\left(r\right)\int_{0}^{\tau +s-r-D}dq\varepsilon_{Y}\left(q\right)\varepsilon_{X}\left(\tau +s-r-q-D\right),$$ (4)

where *D* = 2*d*_*Xd*_+*d*_*Y*_+*d*_*Z*_, and *ε*_*Y*_ and *ε*_*Z*_ are EPSP/IPSP curves of output/inhibitory neurons, respectively. This term primarily causes LTD as the sign flips through lateral inhibition ($-\chi_{2}^{X}(\tau ;w)$; shown as the green line in Fig 1C). Previous simulation studies showed lateral inhibition has critical effects on excitatory STDP learning [17–19]; however, it has not yet been well studied how a secondary correlation generated through the lateral circuits influences STDP at feedforward connections, and it is still largely unknown how lateral inhibition functions with various stimuli in different neural circuits. For example, the correlation kernel of the feedback term exhibits a delay as the signal propagates through the inhibitory circuit; yet, we do not know how much delay is permitted for effective learning or if realistic synaptic delays satisfy such a condition. Furthermore, it is also unknown what information a circuit can learn if there are several mixed signals with different amplitudes for which symmetry-breaking learning [5,39] is not valid. Therefore, using theoretical analysis and simulation, we first investigated the properties of the inhibitory kernel $-\chi_{2}^{X}(\tau ;w)$ in STDP learning.

**Table 1:** Definition of variables.

*s*_*μ*_(*t*)	The activity of external source *μ*	*s*_*μ*_(*t*) = σ[*ν* ^*S*^_*o*_]
*x*_*i*_(*t*)	The spiking activity of input neuron *i*	Eq (14)
*u*_*j*_^*E*^(*t*)	Membrane potential of output neuron *j*	Eq (15)
*y*_*j*_(*t*)	The spiking activity of output neuron *j*	*y*_*j*_(*t*) = σ[*u* ^*E*^_*j*_(*t*)]
*u*_*k*_^*I*^(t)	Membrane potential of inhibitory neuron *k*	Eq (16)
*z*_*k*_(*t*)	The spiking activity of inhibitory neuron *k*	*z*_*k*_(*t*) = σ[*u* ^*I*^_*k*_(*t*)]
*w*_*ji*_^*X*^	The synaptic weight of a feed-forward excitatory connection from *i* to *j*	Eq (17)
*q*_*iμ*_	Response probability of input neuron *i* to external source *μ*	Eq (14)
*C*_*il*_	Non-normalized correlation between input neuron *i* and *l*	*C*_*il*_ = Σ_*μ*_ *q*_*iμ*_ *q*_*lμ*_
*C*_*il*_(*s*)	Cross correlation between input neuron *i* and *l*	Eq (24)
*G*_*1*_^*X*^(*w*), *G*_*2*_^*X*^(*w*)	Coefficients of correlation-based synaptic weight change	Eq (30)
*χ*_*1*_^*X*^, *χ*_*2*_^*X*^	The correlation kernel functions	Eqs (3) and (4)

**Table 2:** Parameter settings.

T	Simulation time	3000 s (for Figs 5C, 5D, 5E, 6 and 7: T = 4000 s)
*L*, *M*, *N*	Neural population	400, 20, 20 (for Figs 7 and 8: M = N = 40)
*L*_*a*_, *M*_*a*_, *N*_*a*_	Neural subpopulation	100, 10, 10
τ_A_^X^, τ_B_^X^, τ_A_^Y^, τ_B_^Y^, τ_A_^Z^, τ_B_^Z^	EPSP/IPSP time constants	5.0, 1.0, 4.0, 0.8, 2.5, 0.5 ms
*w*_*o*_^*X*^, *w*_*o*_^*Y*^, *w*_*o*_^*Z*^	Synaptic weights	2.5, 100.0, 50.0 (for Figs 7 and 8: *w*_*o*_^*Z*^ *= 80*.*0*)
*d* ^Xa^_min_, *d* ^Xa^_max_	Axonal delays	2.0, 4.0 ms
*d* ^Xd^_min_, *d* ^Xd^_max_	Dendritic delays	0.5, 1.5 ms
*d* ^*Y*^_min_, *d* ^*Y*^_max_, *d* ^*Z*^_min_, *d* ^*Z*^_max_	Synaptic (axonal) delays	0.2, 1.2, 0.2, 1.2 ms
θ_t_	Correlation timescale	2.0 ms
*ν*_*o*_^*S*^, *ν*_*o*_^*X*^	Firing rates	10, 10 Hz
*η* ^*X*^	Learning rate	0.05 *w*_*o*_^*X*^
*σ*_*sig*_	Noise amplitude of plasticity	0.3
τ_p_, τ_d_	STDP time windows	17, 34 ms
α, β	Parameters for log-STDP	20.0, 50.0
σ_W_^init^	Initial variance of synaptic weights	0.1
γ^Y^, γ^Z^	LTD/LTP balance	1.4, 0.7

### Lateral inhibition enhances minor source detection by STDP

**Fig 2:**
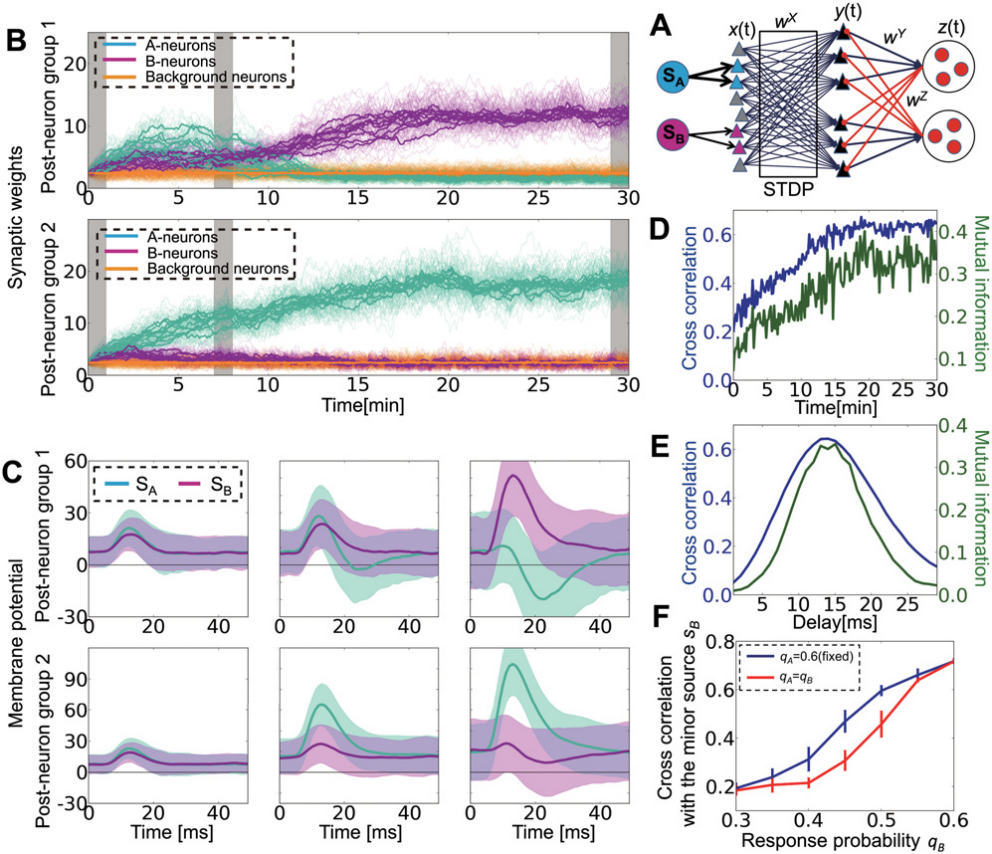
Lateral inhibition enables minor source detection by spike-timing-dependent plasticity (STDP) through membrane hyperpolarization. (A) Schematic figure of the simplified model. S_A_ and S_B_ (on the left side) are the sources that project to subsets of input neurons (colored triangles). Gray triangles are background neurons, black triangles (on the right) are output neurons, and red circles are inhibitory neurons. (B) Development of synaptic weights. Thick lines are mean synaptic weights from A-neurons (blue), B-neurons (red), and Background-neurons (orange) to each output neuron. Thin lines are traces of individual synaptic weights. Gray bar shows the timing at which figure C is calculated. (C) Peristimulus time histograms (PSTHs) of membrane potentials averaged within output neuron groups. T = 0 indicates the timing of events at external layers. The three figures are calculated from the data at t = 0–1 min, 7–8 min, and 29–30 min. (D) Development of mean cross-correlation and mutual information between external sources and population activity of output neurons for the simulation depicted in panels B and C. (E) Delay dependence of mean cross-correlation and mutual information. Both values were calculated from five simulations. (F) Cross-correlation between the output group that detected the minor source and the minor source activity for various response probabilities *q*_*B*_ with a fixed *q*_*A*_ (= 0.6). When none of output groups detected the minor source, the larger value calculated for the two output groups was used. Throughout the study, error bars represent standard deviation calculated from five simulations, unless otherwise indicated.

In Eq (2), if lateral inhibition is negligible (i.e., *g*_*2*_^*X*^ */g*_*1*_^*X*^ = 0), all output neurons acquire the principal component of the response probability matrix *Q*, and the other information is neglected [7,40,41]. On the other hand, if lateral inhibition is effective, different output neurons may acquire various components of the external structure. We first examined that point in a simple network model with two independent external sources (Fig 2A). In the model, each external source drives an independent subgroup of input neurons (we defined those input neurons as A-neurons and B-neurons), which project excitatory inputs to all of the output neurons. Here, we assume that source A drives input neurons with a higher probability than source B (*q*_*A*_ = 0.6, *q*_*B*_ = 0.5), so that input neurons projected by source A show higher correlations (*c*_*A*_ = 0.36) than those receiving the output of source B (*c*_*B*_ = 0.25). In the matrix form,

$$Q=\left(\begin{array}{cc} q_{A} & 0\\ 0 & q_{B}\\ 0 & 0 \end{array}\right),C=\left(\begin{array}{ccc} c_{A} & 0 & 0\\ 0 & c_{B} & 0\\ 0 & 0 & 0 \end{array}\right).$$

The third row in Q represents response probabilities of background neurons in the input layer (gray triangles in Fig 2A; note that C = QQ^t^). We refer to this as the minor source detection task below. Here, for lateral connections, we assumed that both excitatory-to-inhibitory (E-to-I) and inhibitory-to-excitatory (I-to-E) connections are well organized such that inhibition only works mutually between two output neuron groups (Fig 2A; blue lines are E-to-I and red lines are I-to-E connections. See also Eq (30) in Methods). The origin of these structured lateral connections is discussed later. When the network is excited by inputs from external sources, excitatory postsynaptic potential (EPSP) sizes of feedforward connections *W*_*X*_ change according to STDP rules. Initially, in all output neurons, synaptic weights from A-neurons (blue triangles in Fig 2A) become larger because A-neurons are more strongly correlated with one another than B-neurons are. However, as learning proceeds, one of the output neuron groups becomes selective for the minor source B (Fig 2B). After 30 min, the network successfully learns both sources. If we focus on the peristimulus time histogram (PSTH) for the average membrane potential of output neurons aligned to external events, both neuron groups initially show weak responses to both correlation events, and yet the depolarization is relatively higher for source A than for source B (Fig 2C left). After 10 min of learning, both neuron groups show relatively stronger initial responses for source A, but group 1 shows a hyperpolarization soon after the initial response (Fig 2C middle). As a result, synaptic weights from A-neurons to group 1 become weaker, and group 1 neurons eventually become selective for the minor source B (Fig 2C right). The mean cross-correlation (see cross-correlation in Methods for details) between the external sources and the population activity of output neurons is maximized when the delay is approximately 10–15 ms (Fig 2E). If we fix the delay at 14 ms, then the cross-correlation gradually increases as the network learns both sources (Fig 2D). The same argument holds if mutual information is used for performance evaluation (green lines in Fig 2D and 2E). Interestingly, the network better detects the minor source when it is learned with a highly correlated source compared with when it is learned with another minor source (Fig 2F), because a highly correlated opponent source causes strong lateral inhibition on the output neurons, which enhances minor source learning. Similar results are also obtained for conductance-based leaky integrate-and-fire (LIF) neurons (S1 Fig).

### Lateral inhibition should be strong, fast, and sharp

To investigate how and when the network can acquire multiple sources represented by correlated inputs, we further analyzed the model above (see Mean-field approximation of a two-source model in Methods for details). Because both output excitatory neurons and lateral inhibitory neurons are bundled into groups, in the mean-field approximation, we can approximate M excitatory populations and N inhibitory populations into two representative output neurons and two inhibitory neurons. Similarly, input neurons can be bundled into three groups (A-neurons, B-neurons, and Background-neurons). In addition, we assumed that the synaptic connections from Background-neurons to output neurons are fixed because they showed little weight change in the simulation (orange lines in Fig 2B). In this approximation, by inserting Eq (32) into Eq (29), the mean synaptic weight changes of feedforward connections follow

$$\begin{array}{l} \frac{dw_{\mu \nu }^{X}}{dt}≅\sum_{\nu^{\prime }=1}^{L/L_{a}}L_{a}w_{\mu \nu^{\prime }}^{X}\nu_{o}^{S}G_{1}^{X}(w_{\mu \nu }^{X})\sum_{\rho }q_{\nu \rho }q_{\nu^{\prime }\rho }-N_{a}w_{Z}M_{a}w_{Y}\sum_{\nu^{\prime }=1}^{L/L_{a}}L_{a}w_{\bar{\mu }\nu^{\prime }}^{X}\nu_{o}^{S}G_{2}^{X}(w_{\mu \nu }^{X})\sum_{\rho }q_{\nu \rho }q_{\nu^{\prime }\rho }\\ \;+\bar{F}(w_{\mu \nu }^{X})\left[{\left(\nu_{o}^{X}\right)}^{2}\sum_{\nu^{\prime }=1}^{L/L_{a}}L_{a}w_{\mu \nu^{\prime }}^{X}-{\left(\nu_{o}^{X}\right)}^{2}N_{a}w_{Z}M_{a}w_{Y}\sum_{\nu^{\prime }=1}^{L/L_{a}}L_{a}w_{\bar{\mu }\nu^{\prime }}^{X}+{\left(N_{a}w_{Z}\right)}^{2}M_{a}w_{Y}\nu_{o}^{X}\nu_{\mu }^{Z}\right] \end{array},$$ (5)

where *μ* = 1,2 and $\bar{\mu }=2,1$($\mu \neq \bar{\mu }$), and *ν* = A,B. The first two terms are correlation-based learning, and the last term is the homeostatic effect intrinsic to STDP [5]. *G*_*1*_^*X*^ and *G*_*2*_^*X*^ are coefficients determined by synaptic delays, EPSP/IPSP (Inhibitory postsynaptic potential) shapes, and correlation structure, as shown in Eqs (3) and (4). By solving the self-consistency condition (Eq (34) in Methods), the firing rates of inhibitory neurons are approximated as

$$\begin{array}{l} \nu_{1}^{Z}=\frac{M_{a}w_{Y}\nu_{o}^{X}}{1-{\left(M_{a}w_{Y}N_{a}w_{Z}\right)}^{2}}\left[\left(L_{a}w_{1A}+L_{a}w_{1B}+2L_{a}w_{o}^{X}\right)-\left(M_{a}w_{Y}N_{a}w_{Z}\right)\left(L_{a}w_{2A}+L_{a}w_{2B}+2L_{a}w_{o}^{X}\right)\right]\\ \nu_{2}^{Z}=\frac{M_{a}w_{Y}\nu_{o}^{X}}{1-{\left(M_{a}w_{Y}N_{a}w_{Z}\right)}^{2}}\left[\left(L_{a}w_{2A}+L_{a}w_{2B}+2L_{a}w_{o}^{X}\right)-\left(M_{a}w_{Y}N_{a}w_{Z}\right)\left(L_{a}w_{1A}+L_{a}w_{1B}+2L_{a}w_{o}^{X}\right)\right] \end{array}.$$ (6)

**Fig 3:**
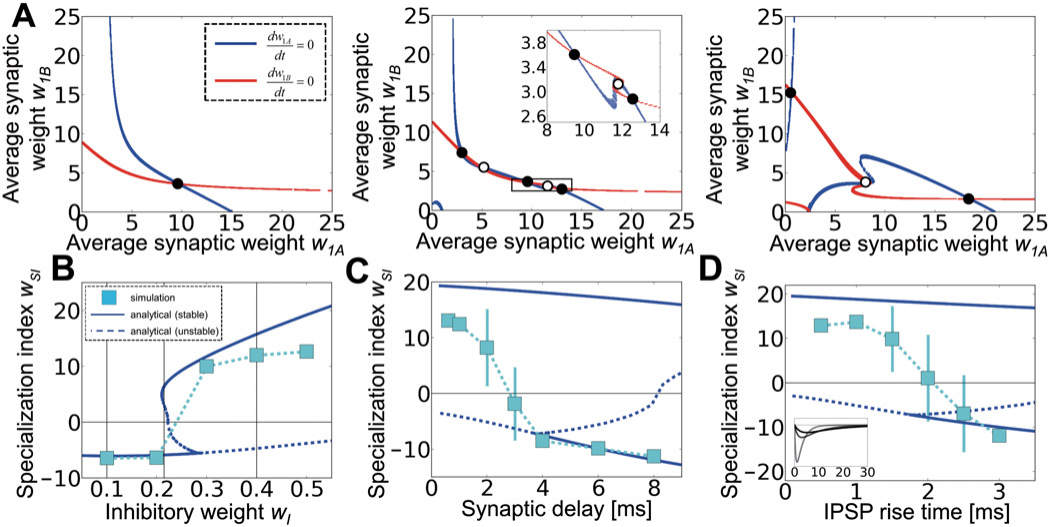
Lateral inhibition is strong, fast, and sharp. (A) Nullclines of the average synaptic weight changes at different inhibitory amplitudes *w*_*Z*_ = 0.1, 0.215, 0.4. The inset in the middle graph is a magnified view of boxed area. (B) Specialization indices *w*_*SI*_ for various inhibitory weights. Positive *w*_*SI*_ indicates the winner-share-all state, whereas negative *w*_*SI*_ indicates the winner-take-all state. Blue lines are analytical estimations and cyan squares are the results of simulations. Vertical lines correspond to the values at which the nullclines in Figure A are calculated. (C) The same graphs for various synaptic delays. The average synaptic delay of both lateral excitatory (*d^Y^_min_*+*d^Y^_max_*)/2 and inhibitory (*d^Z^_min_*+*d^Z^_max_*)/2 connections was changed, while the variability was kept at *d^Y^_max_*—*d^Y^_min_* = *d^Z^_max_*—*d^Z^_min_* = 1.0 ms. (D) IPSP rise time dependence. The inset shows IPSP curves at {τ^Z^_A_, τ^Z^_B_} = {0.5, 2.5} (gray line), {1.5, 7.5} (dark gray line), and {2.5, 12.5} (black line).

We estimated the nullclines by calculating the lines that satisfy ${\dot{w}}_{1\mu }\left(w_{1A},w_{1B},w_{2A}^{*}(w_{1A},w_{1B}),w_{2B}^{*}(w_{1A},w_{1B})\right)=0\;$ for *μ* = *A* or *B*. As a result, we found that when the mutual inhibition is weak (*w*_*I*_ = 10), the system has only one stable point at which *w*_*1A*_ is larger than *w*_*1B*_ (Fig 3A left). At this point, *w*_*2A*_ is also larger than *w*_*2B*_ (*w*_*2A*_ = 9.64, *w*_*2B*_ = 3.60; not shown in the figure), which means that both output neuron groups are specialized for the major source A (we call this state a winner-take-all state or T-state); however, if the inhibition is moderately strong (*w*_*I*_ = 21.5), two new stable fixed points and two unstable fixed points appear in the system (Fig 3A middle). In the stable point on the left, neuron group 1 picks up source B while neuron group 2 picks up source A (*w*_*2A*_ = 12.52, *w*_*2B*_ = 2.87). On the right-hand side, neuron group 1 selects source A while neuron group 2 selects source B (we denote those two states as winners-share-all states or S-states below). At the stable point in the middle, both groups detect source A (*w*_*1A*_ = *w*_*2A*_ = 9.47, *w*_*1B*_ = *w*_*2B*_ = 3.61). Note that because of the mutual inhibition, the synaptic weight from A-neuron is smaller when both groups learn A than it is when only group 1 learns A. For strong inhibition (*w*_*I*_ = 40.0), the stable point in the middle disappears, and the system is stable only when two neuron groups detect different sources (Fig 3A right). Simulation results confirm this analysis because strong inhibition indeed causes a winner-share-all state in which multiple neuron groups survive in competition [15], whereas the network tends to show a winner-take-all learning when the inhibition is weak (Fig 3B). We measured the degree of winner-share-all/winner-take-all states by defining the specialization index *w*_*SI*_ as

$${w^{\prime }}_{SI}=(w_{1A}-w_{1B})(w_{2B}-w_{2A}),w_{SI}={w^{\prime }}_{SI}/\sqrt{\left|{w^{\prime }}_{SI}\right|}.$$ (7)

If *w’*_*SI*_ = 0, we set *w*_*SI*_ = 0. If two output groups are specialized for different sources, *w*_*SI*_ becomes positive, whereas if two groups are specialized for the same source, *w*_*SI*_ becomes negative. When the synaptic delay in the lateral connections is small, only S-states are stable, whereas at longer delays, both S-states and T-states are stable. In the simulation, the network typically grows toward the latter state in the bistable strategy (Fig 3C). Moreover, if we change the shape of the IPSP curve while keeping τ^Z^_B_ = 5 τ^Z^_A_, for steep IPSP curves (i.e., both τ^Z^_A_ and τ^Z^_B_ are small), only the S-states are stable, whereas T-states also become stable for slower IPSPs (Fig 3D). Therefore, both analytical and simulation studies indicate that lateral inhibition should be strong, fast and sharp to detect higher correlation structure. Moreover, lateral inhibition does not need to be pathologically strong because the I/E balance of $N_{a}w_{Z}/Lw_{o}^{X}≅20\%$ is sufficient to cause multistability.

### Optimal correlation timescale changes depend on the noise source

**Fig 4:**
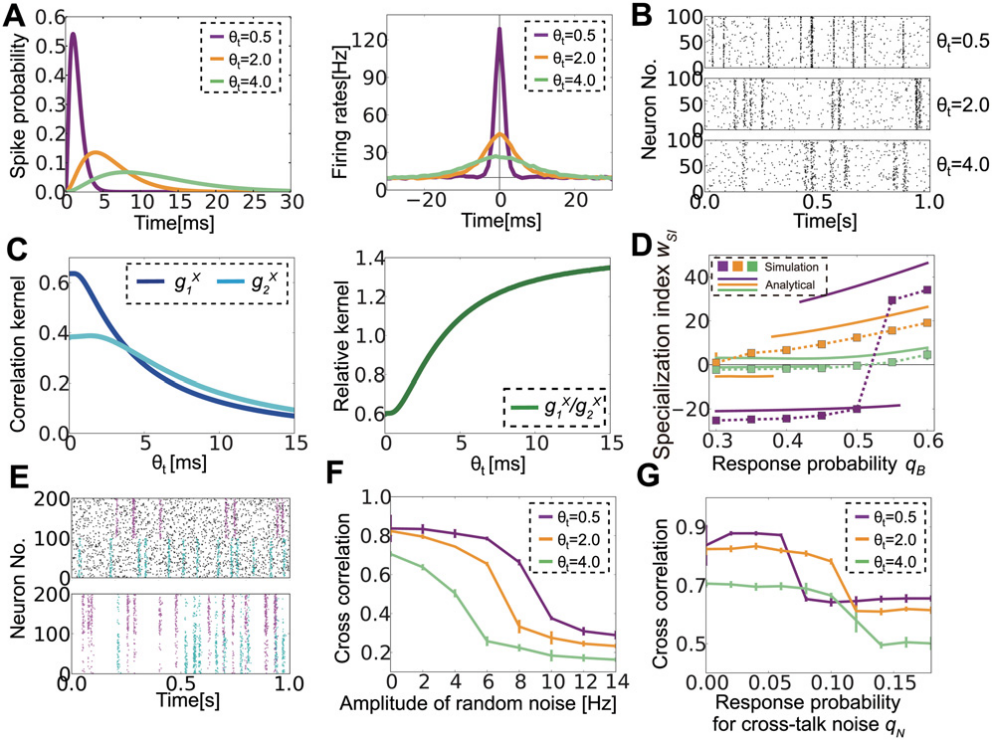
Optimal correlation timescale changes depending on noise characteristics. (A) Response kernels of input neurons to external events (left) and cross-correlation among input neurons responding to the same source calculated from simulated data (right) for three different correlation timescale parameters θ_t_. (B) Raster plots of input neurons for various θ_t_. Only 100 correlated neurons are plotted although there are 400 input neurons in total. (C) Analytically calculated correlation kernels *g*_*1*_^*X*^, *g*_*2*_^*X*^ (left), and their ratio *g*_*1*_^*X*^/*g*_*2*_^*X*^. (D) Specialization index *w*_*SI*_ for various response probabilities *q*_*B*_ while fixing *q*_*A*_ = 0.6. Lines represent *w*_*R*_ at analytically estimated stable points, and dotted squares represent simulation results. (E) Raster plots of two types of noise. The upper panel shows random noise, whereas the lower panel depicts crosstalk noise. In both panels, the first 100 neurons respond primarily to the cyan source, and the next 100 neurons respond to the purple source. For random noise, the noise (black dots) is independent from the signals, whereas the crosstalk noise (purple dots in the lower half, cyan dots in the upper half) is correlated with the signal for the other population. (F, G) The effects of random noise (F) and crosstalk noise (G) at various correlation timescales.

In the previous section, we revealed the effects of network properties for a fixed input correlation structure; however, actual neurons show various timescales for correlations depending on the brain region [37,42] and characteristics of the stimuli [43,44], and it is largely unknown how different timescales influence correlation-driven learning. Therefore, we next considered the effect of correlation timescales, especially on noise tolerance. In our current model, input neurons respond to external sources with input kernel $\phi (t)=t^{2}e^{-t/\theta_{t}}/2\theta_{t}{}^{3}$ (Fig 4A left), and so the correlation between input neuron *i* and *l* is given as

$$C_{il}(s)=\nu_{o}^{S}\sum_{\mu =1}^{p}q_{i\mu }q_{l\mu }h(s).$$

By changing the parameter θ_*t*_, we studied the effect of the correlation timescale on learning. The correlation is precise when θ_*t*_ is small, whereas it becomes broad at large values of θ_*t*_ (Fig 4A right, Fig 4B). Because STDP causes homeostatic plasticity that does not depend on a correlation, as shown in the third term of Eq (5), in a more precise approximation, Eq (2) should be written as

$$\overset{•}{W_{X}}\approx W_{X}\left(g_{1}^{X}E-g_{2}^{X}W_{Z}W_{Y}\right)C^{t}+\langle \text{homeostatic term}\rangle .$$ (8)

We first calculated *g*_*1*_^*X*^ and *g*_*2*_^*X*^ at various θ_t_. Both *g*_*1*_^*X*^ and *g*_*2*_^*X*^ become smaller for a larger θ_t_, but decreases in *g*_*2*_^*X*^ are slower than those in *g*_*1*_^*X*^, and, as a result, *κ* = *g*_*2*_^*X*^/*g*_*1*_^*X*^ becomes larger for a longer correlation timescale (Fig 4C). This means that a longer temporal correlation is more suitable for the detection of multi-components. This is indeed confirmed in the simulation (Fig 4D). When θ_t_ = 0.5 and the minor component is slightly weaker than the major one (*c*_*A*_ = 0.36, *c*_*B*_ = 0.25), the minor component is no longer detectable. On the other hand, at θ_t_ = 2.0, the minor component is detectable even if the strength of the induced correlation is less than half (*c*_*A*_ = 0.36, *c*_*B*_ = 0.16). At θ_t_ = 4.0, *g*_*1*_^*X*^ becomes smaller so that even the major signal is not fully detectable.

Similar results hold for crosstalk noise. In the model above, the noise is provided through the spontaneous Poisson firing of input neurons as random noise (Fig 4E top, black dots are spikes caused by random noise). In reality, however, there would be crosstalk noise among input spike trains caused by the interference of external sources. We implemented this crosstalk noise by introducing non-diagonal components in the response probability matrix as

$$Q=\left(\begin{array}{cc} q_{S} & q_{N}\\ q_{N} & q_{S}\\ 0 & 0 \end{array}\right),$$

where *q*_*S*_ is the response probability to the preferred signal and *q*_*N*_ is that to the non-preferred signal (Fig 4E bottom). We refer to this as the noisy source detection task below. To make a clear comparison, in the simulation of random noise, we kept *q*_*N*_ = 0 and changed the spontaneous firing rate of the input neurons (*r*_*i*_^*o*^) to modify the noise intensity, whereas in simulation of crosstalk noise we removed random noise (i.e., *r*_*i*_^*o*^ = 0) and changed *q*_*N*_. For random noise, a smaller θ_t_ enables better learning because a large *g*_*1*_^*X*^ competes with the homeostatic force (Fig 4F). By contrast, for crosstalk noise, the performance is better at θ_t_ = 2.0 than at θ_t_ = 0.5 because strong lateral inhibition suppresses crosstalk noise (Fig 4G). Although for small noise regimens, the network performs better at θ_t_ = 0.5 than at θ_t_ = 2.0, but the difference is almost negligible. Therefore, to cope with crosstalk noise, the spike correlation needs to be broad, whereas a narrow spike correlation is better for random noise. We note that qualitatively the same arguments as above also hold for the exponential kernel $\phi_{e}(t)=e^{-t/\theta_{t}}/\theta_{t}$ (S3D and S3E Fig). However, the ratio of two coefficients (i.e., *κ*_*e*_ = *g*_*e2*_^*X*^/*g*_*e1*_^*X*^) is typically smaller for this kernel than for the kernel we used throughout this study (S3B and S3C Fig vs. Fig 4D) because lateral inhibition is less effective due to highly peaked spike correlation (S3A Fig).

### Excitatory and inhibitory STDP cooperatively shape structured lateral connections

**Fig 5:**
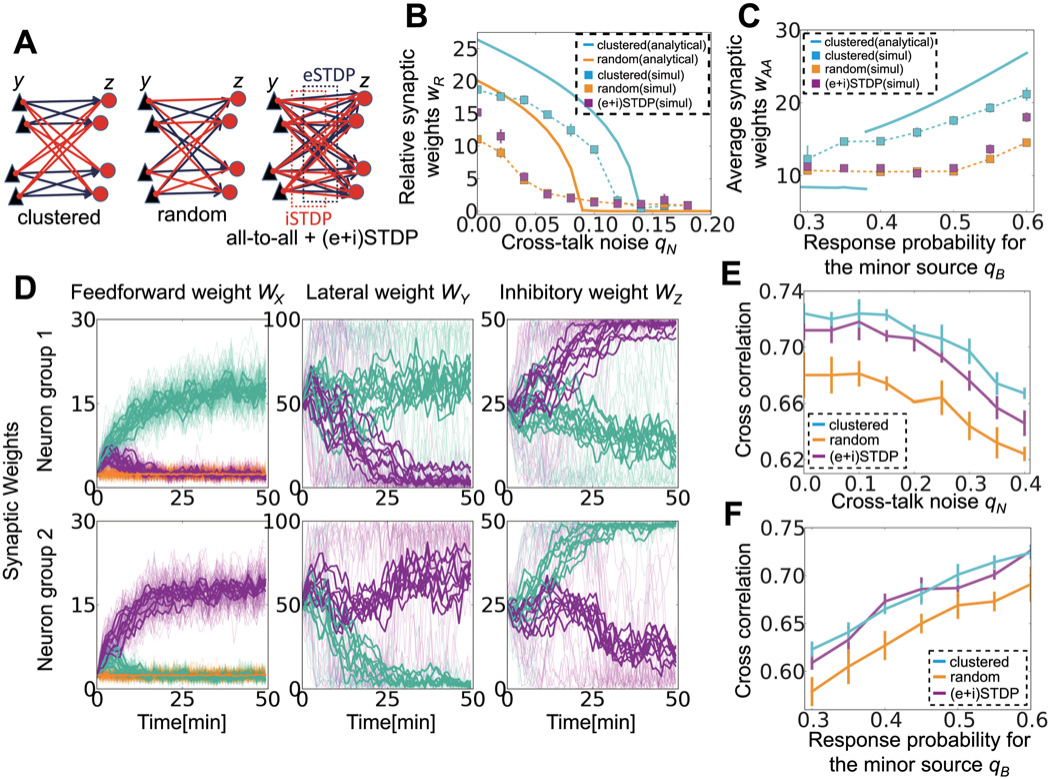
Lateral connection structuring by excitatory and inhibitory spike-timing-dependent plasticity (STDP). (A) Schematic figures of connections between the output layer and the lateral layer. In the simulation, each layer consists of 20 neurons. (B) The effect of crosstalk noise on different lateral structures. Analytical results are shown as bold lines, and the results from simulations are shown as dotted lines. (C) Minor source detection with different lateral structures. Because the specialization index is not well defined for a network with random lateral connections, the average synaptic weights from source A to those output neurons that prefer source A were measured instead. (D) Synaptic weight development at three connections. In the left and right columns, panels show synaptic weights of excitatory/inhibitory synapses projected to the neuron group 1 (top) and group 2 (bottom). In the middle graph, panels correspond to excitatory synapses projected from the neuron group 1 (top) and group 2 (bottom). In all panels, thin lines indicate the development of individual synapses, thick lines represent average weights onto output neurons, and colors indicate A-neurons (blue), B-neurons (red), and Background-neurons (orange). (E, F) Performance of the network with different lateral structures in noisy signal detection (E) and minor signal detection (F). Here (and only here), a pre-learned network is used to investigate responses for various inputs.

To this point, we have considered a network already clustered into two assemblies that inhibit one another (as in Fig 5A left). This means that the network somehow knows a priori that the number of external sources is two; however, in reality, a randomly connected network should also learn such information. To test this idea, we introduced STDP-type synaptic plasticity in lateral excitatory connections and feedback inhibitory connections and investigated how different STDP rules cause different structures in the circuit.

We first checked whether structured lateral connections were helpful for learning. For comparison, we also considered a model with random lateral connections in which all output neurons and inhibitory neurons are randomly connected with probability 0.5 (Fig 5A middle). When lateral connections are random, mean-field equations are modified as

$$\begin{array}{l} \frac{dw_{\mu \nu }^{X}}{dt}≅\sum_{\nu^{\prime }=1}^{L/L_{a}}L_{a}w_{\mu \nu^{\prime }}^{X}\nu_{o}^{S}G^{X}(w_{\mu \nu }^{X})\sum_{\rho }q_{\nu \rho }q_{\nu^{\prime }\rho }-N_{a}w_{Z}M_{a}w_{Y}\sum_{\mu^{\prime }=1}^{p}\sum_{\nu^{\prime }=1}^{L/L_{a}}L_{a}w_{\mu^{\prime }\nu^{\prime }}^{X}\nu_{o}^{S}G^{Y}(w_{\mu \nu }^{X})\sum_{\rho }q_{\nu \rho }q_{\nu^{\prime }\rho }\\ \;+\bar{F}(w_{\mu \nu }^{X})\left[{\left(\nu_{o}^{X}\right)}^{2}\sum_{\nu^{\prime }=1}^{L/L_{a}}L_{a}w_{\mu \nu^{\prime }}^{X}-{\left(\nu_{o}^{X}\right)}^{2}N_{a}w_{Z}M_{a}w_{Y}\sum_{\mu^{\prime }=1}^{p}\sum_{\nu^{\prime }=1}^{L/L_{a}}L_{a}w_{\mu^{\prime }\nu^{\prime }}^{X}+{\left(N_{a}w_{Z}\right)}^{2}M_{a}w_{Y}\nu_{o}^{X}\nu_{tot}^{Z}\right],\\ \nu_{tot}^{Z}\equiv \frac{2M_{a}w_{Y}\nu_{o}^{X}\left(L_{a}w_{1A}+L_{a}w_{1B}+2L_{a}w_{o}^{X}\right)}{1+2M_{a}w_{Y}N_{a}w_{Z}}. \end{array}$$ (9)

We separated lateral connections into two groups as in the previous case, but this approximation is legitimate only when two input sources are symmetrical (i.e., *q*_*A*_ = *q*_*B*_). In other cases, neurons are often organized into two groups with different population sizes. In such cases, for evaluating performance, we measured average weights from source A on the output neurons receiving stronger inputs from A-neurons than from B-neurons or Background-neurons. For randomly connected lateral inhibition, learning performance dropped significantly in noisy source detection (Fig 5B) and in minor source detection (Fig 5C); thus clustered connectivity is indeed advantageous for learning.

We next investigated whether such structure can be learned using STDP rules. We first introduced Hebbian STDP for both E-to-I and I-to-E connections. With these learning rules, the lateral connections successfully learn a mutual inhibition structure (Fig 5D); however, this learning is achievable only when the learning of a hidden external structure is possible from the random lateral connections (magenta lines in Fig 5B and 5C; note that orange points are hidden by magenta points because they show similar behaviors in noisy cases), which means either when crosstalk noise is low or two sources have similar amplitudes. Nevertheless, once a structure is obtained in easy settings (*q*_*N*_ = 0 or *q*_*A*_ = *q*_*B*_), that network outperforms the network with random lateral connections in both noisy source detection (Fig 5E) and minor source detection (Fig 5F). In Fig 5E, we evaluated the performance of noisy source detection by first conducting STDP learning at *q*_*N*_ = 0, and then we terminated STDP and performed simulations at the various noise levels *q*_*N*_. Similarly, in the minor source detection task depicted in Fig 5F, we first performed STDP learning with *q*_*A*_ = *q*_*B*_ = 0.6, and then evaluated the performance for a smaller *q*_*B*_. STDP can also generate similar lateral connection structures when the total number of input sources is larger than two (S2A and S2B Fig). Therefore, STDP at lateral connections helps signal detection by efficiently organizing the connection structure.

We next studied the analytical conditions for learning of the clustered structure (see Analytic approach for STDP in lateral and inhibitory connections in Methods for details). The synaptic weight dynamics of lateral excitatory and inhibitory connections are approximately given as

$$\begin{array}{l} \overset{•}{W_{Y}}\approx g_{1}^{Y}W_{Y}W_{X}C^{t}W_{X}^{t},g_{1}^{Y}\equiv \int_{-\infty }^{\infty }dsF^{Y}(s)\int D_{r}^{X}\int D_{u}^{Y}\int D_{r^{\prime }}^{X}h(u+r^{\prime }-s-r)\\ \overset{•}{W_{Z}}\approx g_{1}^{Z}W_{X}CW_{X}^{t}W_{Y}^{t},g_{1}^{Z}\equiv \int_{-\infty }^{\infty }dsF^{Z}(s)\int D_{r}^{X}\int D_{u}^{Y}\int D_{r^{\prime }}^{X}h(r-s-u-r^{\prime }-d_{Z}-d_{Y}). \end{array}$$ (10)

**Fig 6:**
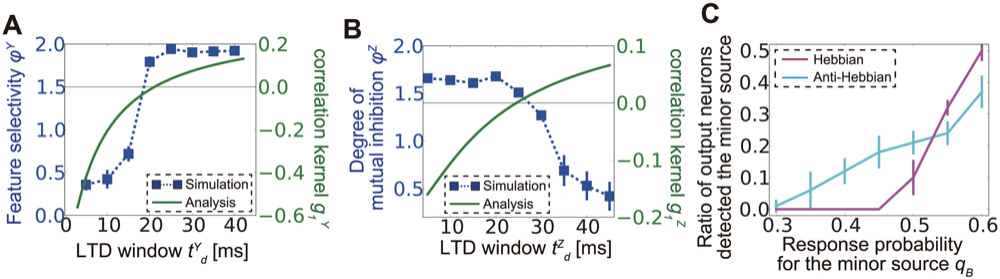
Correlation propagation shapes lateral connection structure. (A) Comparison between feature selectivity ϕ^*Y*^ (blue dots) calculated from simulation results and analytically calculated correlation kernel function *g*_*1*_^*Y*^ (green line) for lateral excitatory connections. Thin green horizontal line represents *g*_*1*_^*Y*^ = 0. (B) Comparison between the degree of mutual inhibition ϕ^*Y*^ (blue dots) calculated from the simulation and analytically calculated correlation kernel *g*_*1*_^*Z*^ (green line) for lateral inhibitory connections. Negative *g*_*1*_^*Z*^ is correlated with a high degree of mutual inhibition, as expected (see Methods). (C) Ratio of output neurons tuned for the minor source in a minor source detection task under Hebbian and anti-Hebbian inhibitory spike-timing-dependent plasticity.

Both equations represent indirect effects of the input correlation propagated into the lateral circuit. From a linear analysis, we can expect that when *g* ^*Y*^_*1*_ is positive, E-to-I connections tend to be feature selective (see Eq (35) in Methods). Each inhibitory neuron receives stronger inputs from one of the output neuron groups and, as a result, shows a higher firing rate for the corresponding external signal. On the other hand, if *g* ^*Z*^_*1*_ is positive, I-to-E connections are organized in reciprocal form, where one of the reciprocal connections is enhanced and the other is suppressed (see Eq (36) in Methods). We can evaluate feature selectivity of inhibitory neurons by

$$\varphi^{Y}=\frac{1}{N}\overset{N}{\underset{k=1}{\sum }}\left(\frac{1}{\left|\Omega_{A}^{Y}\right|}\underset{j\in \Omega_{A}^{Y}}{\sum }w_{kj}^{Y}-\frac{1}{\left|\Omega_{B}^{Y}\right|}\underset{j\in \Omega_{B}^{Y}}{\sum }w_{kj}^{Y}\right)/\left(\frac{1}{M}\overset{M}{\underset{j=1}{\sum }}w_{kj}^{Y}\right),$$ (11)

where Ω^Y^_A_ and Ω^Y^_B_ are the sets of excitatory neurons responding preferentially to sources A and B, respectively. Indeed, when the LTD time window is narrow, analytically calculated *g* ^*Y*^_*1*_ tends to take negative values (the green line in Fig 6A), and E-to-I connections organized in the simulation are not feature selective (the blue points in Fig 6A). By contrast, for a long LTD time window (i.e., when LTD is weakly spike-timing dependent), *g* ^*Y*^_*1*_ tends to take positive values, and E-to-I connections become clustered. In the simulation, *W*_*Z*_ is also plastic, but as shown in Eq (10), the effect of *W*_*Z*_ on the plasticity of *W*_*Y*_ is negligible in first-order approximations.

Similarly, for I-to-E connections, we measure the degree of mutual inhibition (non-reciprocity) with

$$\varphi^{Z}=\frac{1}{N}\sum_{k=1}^{N}\left|\frac{w_{kj}^{Y}}{\sum_{j=1}^{M}w_{kj}^{Y}}-\frac{w_{jk}^{Z}}{\sum_{j=1}^{M}w_{jk}^{Z}}\right|$$ (12)

When LTD is strongly spike-timing dependent, *g* ^*Z*^_*1*_ is negative and ϕ^Z^ calculated from the simulation data tends to be large (Fig 6B), which means that inhibitory connections are organized such that the inhibition functions as mutual inhibition between excitatory neuron groups. Note that the organized neuronal wiring patterns are not a pure product of the pre-post causality of STDP but the effect of spike correlations propagating through lateral inhibitory circuits. If the structural plasticity is merely caused by the pre-post causality, both ϕ^Y^ and ϕ^Z^ can decrease with increases in the inhibitory population while maintaining the total synaptic weights because the causal effect becomes weaker as each synaptic weight becomes smaller [45]; however, in our simulations, the values of both quantities generally increased for larger inhibitory populations (S2C Fig).

Hebbian inhibitory STDP at lateral connections is not always beneficial for learning. For example, in minor source detection, if we use Hebbian inhibitory STDP, a slightly minor source is not detectable, whereas for anti-Hebbian STDP, a small number of neurons still detect the minor source because reciprocal connections from strong-source responsive inhibitory neurons to strong-source responsive output neurons inhibit synaptic weight development for the stronger source (Fig 6C).

### Neural Bayesian ICA and blind source separation

**Fig 7:**
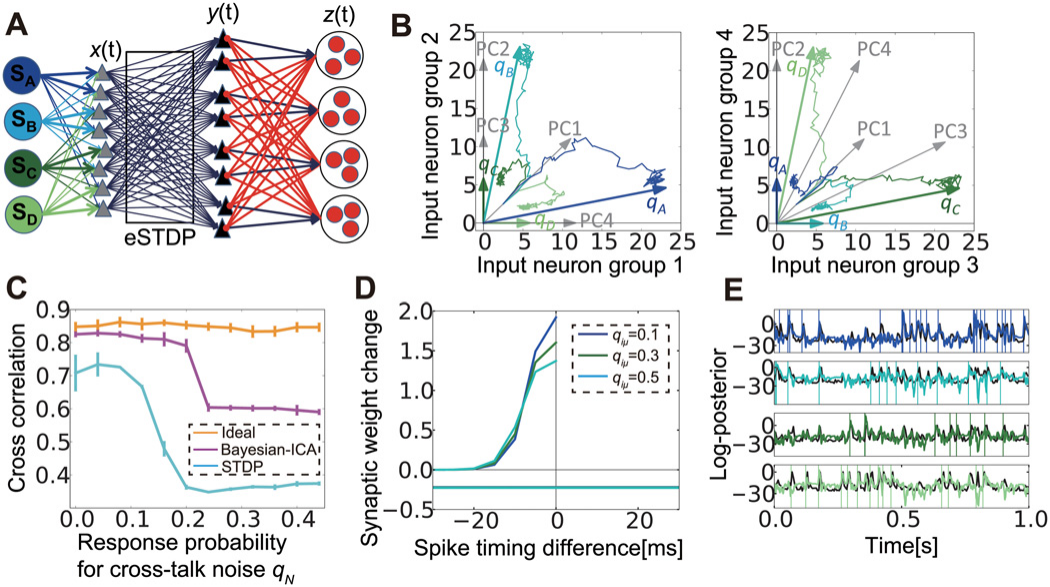
With lateral inhibition, spike-timing-dependent plasticity (STDP) mimics Bayesian independent component analysis (ICA). (A) Schematic figure of the model with four sources. (B) Synaptic weight development in input neuron space. Arrows *q*_*A*_ to *q*_*D*_ are response probability vectors of the four sources, and PC1 to PC4 are normalized principal components of the correlation matrix C. Lines represent traces of average synaptic weight from each input group to the output groups that learned corresponding sources during the learning process. (C) Comparison of performance among the ideal observer, Bayesian ICA learning, and STDP learning. (D) LTP/LTD time window of Bayesian ICA learning. (E) Behaviors of log-membrane potential (color lines) in the STDP model, and estimated log-posterior (black lines) in the Bayesian ICA algorithm for the same stimuli. Vertical lines represent timings of external events. Log-membrane potentials are normalized to align the mean and the variance to the corresponding log-posteriors.

Our results to this point have revealed that correlation-based STDP learning combined with lateral inhibition can successfully detect signals from mixed inputs masked by noises. To confirm this mechanism is indeed effective in realistic tasks, we applied the above method to blind source separation. We first examined the condition in which the network could capture external sources. We extended the previous network to include four independent sources mixed at the input layer (Fig 7A). In the present application, we used structured lateral connections because learning for clustered structures is difficult with noisy stimuli, as shown in the preceding section. The response probability matrix Q and correlation matrix C are given as

$$Q=\left(\begin{array}{cccc} q_{S} & q_{N} & 0 & q_{N}\\ q_{N} & q_{S} & q_{N} & 0\\ 0 & q_{N} & q_{S} & q_{N}\\ q_{N} & 0 & q_{N} & q_{S} \end{array}\right),\;C=(\begin{array}{cccc} q_{S}{}^{2}+2q_{N}{}^{2} & 2q_{S}q_{N} & 2q_{N}{}^{2} & 2q_{S}q_{N}\\ 2q_{S}q_{N} & q_{S}{}^{2}+2q_{N}{}^{2} & 2q_{S}q_{N} & 2q_{N}{}^{2}\\ 2q_{N}{}^{2} & 2q_{S}q_{N} & q_{S}{}^{2}+2q_{N}{}^{2} & 2q_{S}q_{N}\\ 2q_{S}q_{N} & 2q_{N}{}^{2} & 2q_{S}q_{N} & q_{S}{}^{2}+2q_{N}{}^{2} \end{array}).$$

Therefore, the principal components of matrix Q (i.e., eigenvectors of C) are {1, 1, 1, 1,}, {-1, 0, 1, 0}, {0, -1, 0, 1}, {-1, 1, -1, 1}. Because the first-order approximation of synaptic weight dynamics follows $\overset{•}{W_{X}}\approx g_{1}^{X}W_{X}C^{t}$, we may expect that synaptic weight vectors converge to the eigenvectors of the principal components; however, this was not the case in our simulations, even if we took into account the non-negativity of synaptic weights (see Fig 7B, where we renormalized the principal vectors to the region between 0 and 1). Instead, each weight vector converged to a column of the response probability matrix Q (Fig 7B, the left panel is the projection to the first two dimensions, and the right panel is the projection to the other two dimensions). This result implies that the network can extract independent sources, rather than principal components, from multiple intermixed inputs.

We next evaluated the performance of hidden external source detection, especially its tolerance against crosstalk noise. To this end, we compared the performance of the model with that of the Bayesian ICA algorithm, in which independence of external sources is treated as a prior [46,47]. In the algorithm, the learned mixing matrix may converge to its Bayesian optimal value estimated from a stream of inputs. Although we cannot directly argue the optimality of cross-correlations, if the mixing matrix is accurately estimated, external activity is also well inferred, and thus we can use the mean cross-correlation as a measure for the optimality of learning. In terms of discretized input activity *X*, the external source activity *S* and prior information *I*, we can express the conditional probability of the estimated response probability matrix $\tilde{Q}$ as $P\left[\tilde{Q}|X,I\right]=\frac{P\left[\tilde{Q}|I\right]}{P\left[X|I\right]}\int P\left[X|S,\tilde{Q},I\right]P\left[S|I\right]dS$ (see Bayesian ICA in Methods for details). This means that even if no prior information is given for $\tilde{Q}$ itself (i.e.$P\left[\tilde{Q}|I\right]=const.$), posterior $P\left[\tilde{Q}|X,I\right]$ still depends on a prior given for *S*. If we introduce a prior that each external source follows an independent Bernoulli Process (i.e.$P\left[S|I\right]=\prod_{k=1}^{T/\Delta t}\prod_{i=1}^{L}{\left(r_{s}\Delta t\right)}^{s_{\mu }^{k}}{\left(1-r_{s}\Delta t\right)}^{1-s_{\mu }^{k}}$), then the stochastic gradient descendent of posterior function is given as,

$$\frac{\partial }{\partial {\tilde{q}}_{i\mu }}\log P\left[\tilde{Q}|X,I\right]=\frac{1}{Z_{p}}\sum_{k=1}^{T/\Delta t}\int P[S,X|\tilde{Q},I]\frac{2x_{i}^{k}-1}{x_{i}^{k}p_{i}^{k}/\left(1-p_{i}^{k}\right)+(1-x_{i}^{k})}\frac{\sum_{k^{\prime }=0}^{\infty }\phi_{k^{\prime }}s_{\mu }^{k-k^{\prime }}}{1-{\tilde{q}}_{i\mu }\sum_{k^{\prime }=0}^{\infty }\phi_{k^{\prime }}s_{\mu }^{k-k^{\prime }}}dS,$$

where

$$p_{i}^{k}=1-\left(1-r_{i}^{o}\Delta t\right)\prod_{\mu =1}^{p}\left[1-{\tilde{q}}_{i\mu }\sum_{k^{\prime }=0}^{\infty }\phi_{k^{\prime }}s_{\mu }^{k-k^{\prime }}\right],\phi_{k}=\frac{1}{2\theta_{t}{}^{3}}{\left[\left(k+1/2\right)\Delta t\right]}^{2}\exp\left[-\left(k+1/2\right)\Delta t/\theta_{t}\right].$$

We approximated this Bayesian ICA algorithm by a sequential sampling source activity instead of calculating the integral over all possible combinations in the estimation of the log-posterior of the response probability matrix Q. In this approximation, the learning rule of the estimated response probability matrix $\tilde{Q}$ obeys

$$\Delta {\tilde{q}}_{i\mu }^{k}\propto \frac{2x_{i}^{k}-1}{x_{i}^{k}p_{i}^{k}(Y^{1:k-1})/\left(1-p_{i}^{k}(Y^{1:k-1})\right)+(1-x_{i}^{k})}\times \frac{\sum_{k^{\prime }=0}^{\infty }\phi_{k^{\prime }}y_{\mu }^{k-k^{\prime }}}{1-{\tilde{q}}_{i\mu }^{k}\sum_{k^{\prime }=0}^{\infty }\phi_{k^{\prime }}y_{\mu }^{k-k^{\prime }}}p_{i}^{k}(Y^{1:k-1})=1-\left(1-r_{i}^{o}\Delta t\right)\prod_{\mu =1}^{p}\left[1-{\tilde{q}}_{i\mu }^{k}\sum_{k^{\prime }=0}^{\infty }\phi_{k^{\prime }}y_{\mu }^{k-k^{\prime }}\right],$$ (13)

where Y is the sampled sequence, and *p*_*i*_^*k*^(Y^1:k-1^) is the sample based approximation of p_i_^k^ in the previous equation. This rule has spike-timing and weight dependence similar to those seen in STDP (Fig 7D). Although the performance of STDP is much worse than the ideal case (when the true Q is given), this performance is similar to that for the sample-based learning algorithm discussed above (Fig 7C). Therefore, the network detects independent sources if crosstalk noise is not large. We further studied the response of the models for the same inputs and found that the logarithm of the average membrane potential $u_{\mu }^{E}=\frac{1}{\left|\Omega_{\mu }\right|}\sum_{j\in \Omega_{\mu }}u_{j}^{E}$ well approximates the log-posterior estimated in Bayesian ICA, even in the absence of a stimulus (Fig 7E). This result suggests that in the STDP model, expected external states are naturally sampled through membrane dynamics that are generated through the interplay of feedforward and feedback inputs.

**Fig 8:**
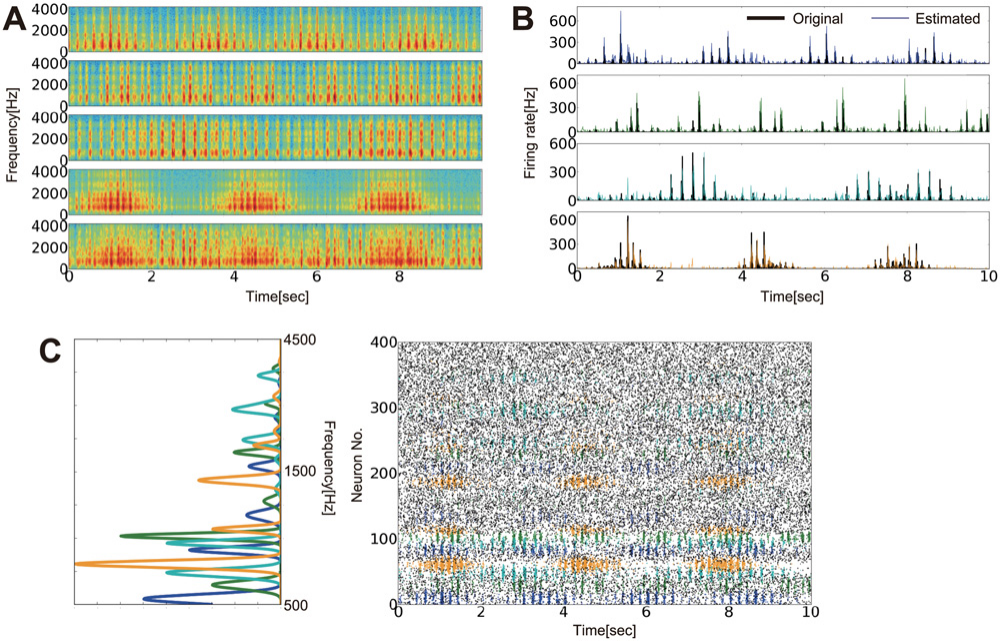
Blind source separation by spike-timing-dependent plasticity (STDP). (A) Four original auditory signals (from the top to the fourth set of signals) and one mixed signal (bottom). (B) Amplitudes of original signals (black lines) and those estimated from output firing rates (colored lines). (C) Spectra of auditory sources *a* ^*q*^_*h*_(*f*) (left). Raster plots of input neuron activity. Colors were probabilistically assigned based on expected sources. All figures were calculated from the 30’00”–30’10” portion of the auditory signals and simulation.

We finally performed the blind separation task using the same network as shown in Fig 7A. We created “sensory” inputs by mixing four artificially created auditory sequences (Fig 8A and S1 Auditory File). In the auditory cortex, various frequency components of a sound, particularly high-frequency components, are represented by specific neurons typically organized in a tonotopic map structure [48], whereas low-frequency components are expected to be perceived as a change in sound pressure. Furthermore, populations of neurons in the primary auditory cortex are known to synchronize the relative timing of their spikes during auditory stimuli and provide correlated spike inputs for higher cortical areas in which the auditory scene is fully analyzed and perceived [49,50]. We modeled these features by assuming that input neurons have a preferred frequency {*f*_*i*_} defined as

$$f_{i}=\exp\left[\frac{i}{L}\left(\log f_{\max}-\log f_{\min}\right)+\log f_{\min}\right],$$

and auditory inputs are provided as time-dependent response probabilities, which follow $q_{i}(t)=q_{o}\sum_{q}a_{l}^{q}(t)a_{h}^{q}(f_{i})$, where *a* ^*q*^_*h*_(*f*) is the spectrum of auditory source *q* (left panel of Fig 8C), and *a* ^*q*^_*l*_(t) is the temporal change of the sound pressure (black lines in Fig 8B). In this representation, each sound source is represented by correlated spikes of neural populations (right panel of Fig 8C). Even if signals have overlapping frequency components {*a* ^*q*^_*h*_(*f*)}_*q*_, blind separation is possible as long as {*a* ^*q*^_*l*_(*t*)}_q_ are independent and have sharp rising profiles sufficient to cause spike correlations. After learning, four output neuron groups successfully detected changes in the sound pressure of the four original auditory signals (colored lines in Fig 8B) by correctly identifying the input neurons that encoded the signals. Therefore, STDP rules implemented in a feedforward neural network with lateral inhibition serve as a spike-based solution to the blind source separation or cocktail party effect problem.

## Discussion

By analytically investigating the propagation of input correlations through feedback circuits, we revealed how lateral inhibition influenced plasticity at feedforward connections. We showed that a population of neurons could learn multiple signals with different strengths or mixed levels. In addition, we found that to perform learning from signals corrupted with random noise, the timescale of the input correlations needed to be in the range of milliseconds, whereas the timescale was broader for crosstalk noise, which may explain why the spike correlation of cortical neurons often exhibits a large jitter (approximately 10 ms) [36,37]. We also investigated the functional roles of STDP at lateral excitatory and inhibitory connections to demonstrate that Hebbian STDP shaped the lateral structure to improve signal detection performance. Our results also suggested that anti-Hebbian plasticity was helpful for learning from minor sources and implied that different STDP rules at lateral connections induced different algorithms at feedforward connections. Furthermore, we derived an STDP-like online learning rule by considering an approximation of Bayesian ICA with sequence sampling. This result suggested that lateral inhibition adjusted the membrane potentials of postsynaptic neurons so that their spiking processes accurately performed sequence sampling. We also demonstrated that this mechanism was applicable to blind source separation of auditory signals.

### Noise characteristics and correlation timescales

Simultaneously recorded neurons in close proximity often show correlated spiking, yet the precision of these correlations varies across brain regions. Neurons in the lateral geniculate nucleus show strong spike correlations [42,51], while correlations in V1 [36,52] or higher visual areas [37] are less precise. Our results indicate the interesting possibility that these differences may reflect the different characteristics of the noise with which the various cortical areas need to contend. At an early stage of sensory processing, the major noise component may be environmentally produced background noise from various sources; thus precise spike correlation is beneficial at this stage for noise reduction during signal detection and learning (Fig 4G). By contrast, in higher sensory cortices, crosstalk noise accumulated through signal propagation in circuits may form the primary noise source, so less precise spike correlation is preferable (Fig 4H). It would be intriguing to examine whether lower and higher cortical areas similarly change the strength of spike correlations for other sensory modalities.

### STDP in E-to-I and I-to-E connections

It is known that both glutaminergic synapses on inhibitory neurons [53,54] and GABAergic synapses on excitatory neurons [55,56] show STDP, and it is also known that STDP at E-to-I connections plays an important role in developmental plasticity [57]; however, detailed properties of these plasticities are still largely disputable [58,59] and, reportedly, highly dependent on inhibitory cell type [60], neuromodulator [61], and region [58]. We showed that in a feedback circuit, Hebbian inhibitory STDP preferred winner-take-all while anti-Hebbian inhibitory STDP tended to cause winner-share-all (see Fukai and Tanaka 1997 for winner-share-all) at excitatory neurons (Fig 6D). This result indicates that different inhibitory STDP imposes different functions for excitatory STDP, which suggests that a neural circuit may select optimal inhibitory STDP for a specific purpose or strategy of learning, and this may differ across regions and be modified by neuromodulators. A recent study showed that inhibitory plasticity even directly influences the plasticity at excitatory synapses of the postsynaptic neuron [62]. In such cases, algorithm selection would play a more important role than it did for the standard STDP implemented in our model.

Recently, inhibitory neurons in the rodent hippocampus CA1 were shown to display context-dependent activity rate changes during a spatial learning task, in association with the activity rate changes in excitatory cells [63]. In addition, the authors suggested the candidate mechanism for this change in activity is STDP at E-to-I synapses. Our results examining E-to-I STDP confirmed this configuration of inhibitory cells modulated by plasticity at feedforward excitatory connections (Fig 5D, S2A and S2B Fig). In our model, although inhibitory neurons are not directly projected from input sources, as excitatory neurons learn a specific input source (Fig 5D, left panel), inhibitory neurons acquire feature selectivity through Hebbian STDP at synaptic connections from those excitatory neurons (Fig 5D, middle panel). Furthermore, our results indicate an important function of these feature-selective inhibitory neurons. Once an adequate circuit structure is learned and inhibitory connections are organized into a feature-selective pattern, even if the input to the network becomes noisy or faint, the network can still robustly detect signals (Fig 5E and 5F). This robustness would be useful for spatial learning, as contextual information is often uncertain.

### STDP and Bayesian ICA

Our results indicated that STDP in a lateral inhibition circuit mimicked Bayesian ICA [46,47]. First, output neurons were able to detect hidden external sources, without capturing principal components (Fig 7B). Previous results suggest that for a single output neuron, an additional homeostatic competition mechanism is necessary to detect an independent component [7,22]. In addition, when information is coded by firing rate, homeostatic plasticity is critically important, because STDP itself does not mimic Bienenstock-Cooper-Munro learning [18]. However in our model, information was encoded by correlation, and mutual inhibition naturally induced intercellular competition so that intracellular competition through homeostatic plasticity was unnecessary. Moreover, our analytical results suggested the reason that independent sources are detected. To perform a principal components analysis using neural units, the synaptic weight change needs to follow

$$\overset{•}{W_{X}}=W_{X}C-LT[W_{X}CW_{X}^{t}]W_{X},$$

where LT[] means lower triangle matrix [64,65]. This LT transformation protects principal components caused by the lateral modification from higher order components; however in our model, because all output neurons receive the same number of inhibitory inputs Eq (2), all neurons are decorrelated with one another and develop into independent components.

Recently, it was shown that STDP can perform Bayesian optimal learning [66,67]. In the model used by those authors, the synaptic weight matrix is treated as a hyper parameter and estimated by considering the maximum likelihood estimation of input spike trains. By contrast, in the Bayesian ICA framework, the mixing matrix (corresponding to synaptic weight matrix) is treated as a probabilistic variable. Using this framework, we needed to calculate an integral over all possible source activities in the past to derive stochastic gradient descendent; however, as shown in Fig 7C, the stochastic learning was well performed by employing an approximation with sequential sampling. Moreover, we naturally derived an adequate LTP time window from the response kernel of input neurons to external events (Fig 7D). We also found that STDP self-organized a lateral circuit structure that performed better than a random global inhibition (Fig 5E and 5F). Mathematically, to perform sampling from a probabilistic distribution, we first needed to calculate the occurrence probability of each state; however, in a neural model, membrane potentials of output neurons approximately represent the occurrence probability through membrane dynamics. In machine learning methods, integration over possible source activities is often approximated using Markov chain Monte Carlo (MCMC) sampling [68]. Interestingly, a recent study showed that a recurrent network performed MCMC sampling [69,70], suggesting that our network may perform a more accurate sampling in the presence of recurrent excitatory connections.

### Suboptimality of STDP

Previous theoretical results suggest that STDP can modulate synaptic weights in a way that optimizes information transmission between pre- and postsynaptic neurons [71,72]. In the Bayesian ICA framework, blind source separation can be formulized as an optimization problem, but, in this case, the problem itself is ill-defined because optimality does not guarantee the true solution. In addition, local minima are often unavoidable for online learning rules. Nevertheless, the problems faced by the brain are often ill-defined, and suboptimality is inevitable [73]. Because we performed both nonlinear dynamics-based and machine learning-based analyses, we can offer some insights regarding the origins of local minima in stochastic gradient descendent learning. In the initial state, synaptic weights are typically homogeneously distributed, and this state is often locally stable. As a result, the homogeneous stable point is more likely to be selected in learning (Fig 2C and 2D) than the non-homogenous, more desirable, points; however, introducing additional noise may change this situation. Indeed, in Fig 4B and Fig 7C, the performance of the model was improved by adding a small amount of noise to input activities, although the improvement was not significant; however, because a large amount of noise is harmful for computations and stable learning, the benefit of noise addition is highly limited, and the brain may recruit other mechanisms for near optimal learning.

### Neural mechanism of blind source separation

Humans and nonhuman animals can detect a specific auditory sequence from a mixed, noisy auditory stimulus, a phenomenon often called the cocktail party effect. The mechanism underlying the cocktail party effect remains elusive [26,28,29], although several solutions have been proposed [74,75]. An effective solution for this problem is ICA [76–78], and the neural implementation of the algorithm has been studied by several authors [14,18,79,80]. Our study extended these results through a rigorous analytical treatment on biologically plausible STDP learning of spiking neurons, and our analyses enabled us to discover interesting functions of correlation coding. Moreover, by explicitly modeling inhibitory neurons, we found that STDP at E-to-I and I-to-E connections cooperatively organized a lateral structure suitable for blind source separation. In addition, we successfully extended a previous model for the formation of static visual receptive fields [18,19] to a more dynamic model in an auditory blind source separation task. In realistic auditory scene analysis, the frequency spectrum of acoustic signals is first analyzed in the cochlea, where each frequency component is the mixture of sound components from independent sources. Components belonging to the same source may be separated and integrated by downstream auditory neurons for the perception of the original signal. These frequency components can be considered a mixed signal in the ICA problem [81]; thus even if signals are mixed in frequency space, if the amplitudes of the signals are temporally independent, blind separation is still achievable. In the neural implementation of the problem, if two frequencies are commonly activated in the same signal, neurons representing those frequencies show spike correlation under the presence of the signal; thus the learning process is naturally achieved by STDP learning. These results indicate an active role of spike correlation and STDP in efficient biological learning.

## Methods

### Model

#### Neural dynamics

Based on the previous study [7], we constructed a network model with one external layer and three layers of neurons (Fig 1A). The first layer is the external layer that corresponds to external stimuli or the sensory system’s response to these stimuli. For simplicity, we approximated the activity of external sources using a Poisson process with the constant rate *ν* ^*S*^_*o*_. If we define the Poisson process with rate *r* as $\hat{\sigma }(r)$, the activity of the external source *μ* at time *t* is written as $s_{\mu }(t)=\hat{\sigma }(\nu_{o}^{S})$ (see Table 1 for the list of variables). Neurons in the input layer fire spikes in response to activity in the external layer. By assuming a rate-modulated Poisson process, the spiking activity of the input neuron *i* follows

$$x_{i}(t)=\hat{\sigma }\left[r_{i}^{o}+\sum_{\mu =1}^{p}q_{i\mu }\int_{0}^{\infty }\phi (t^{\prime })s_{\mu }(t-t^{\prime })dt^{\prime }\right],$$ (14)

where *r*_*i*_^*o*^ is the instantaneous firing rate defined with $r_{i}^{o}=\nu_{o}^{X}-\sum_{\mu =1}^{p}q_{i\mu }\nu_{o}^{S}$, *q*_*iμ*_ is the response probability for the hidden external source *μ*, and $\phi (t)=t^{2}e^{-t/\theta_{t}}/2\theta_{t}{}^{3}$ is the response kernel for each external event. In most theoretical studies, cross-correlations give an exponential decay or a delta function [5,38], but here we used a response kernel that produces broader correlations (Fig 4A right), because the actual correlations observed in the cortex are usually not sharply peaked [36,37]. For instance, for the exponential kernel $\phi_{e}(t)=e^{-t/\theta_{t}}/\theta_{t}$, correlations show a peaked distribution even if the timescale parameter θ_t_ is several milliseconds (S3A Fig). Because of the common inputs from the external layer, input neurons show highly correlated activity, which enables population coding of the hidden structure. Although here we explicitly assumed the presence of the external layer, these analytical results can also be applied for arbitrary realization of a spatiotemporal correlation.

Output neurons are modeled with the Poisson neuron model [5,38,45] in which the membrane potential of neuron *j* at time *t* is described as

$$u_{j}^{E}(t)=\sum_{i=1}^{M}w_{ji}^{X}\int_{0}^{\infty }\varepsilon_{X}(r)x_{i}(t-r-d_{ji}^{X})dr-\sum_{k=1}^{N}w_{jk}^{Z}\int_{0}^{\infty }\varepsilon_{Z}(r)z_{k}(t-r-d_{jk}^{Z})dr,$$ (15)

where *w*_*ji*_^*X*^ and *w*_*jk*_^*Z*^ are the EPSPs/IPSPs of input currents from input neuron *x*_*i*_ and lateral neuron *z*_*k*_, respectively, convolution functions are defined as $\varepsilon_{X}(r)=\frac{e^{-r/\tau_{A}^{X}}-e^{-r/\tau_{B}^{X}}}{\tau_{A}^{X}-\tau_{B}^{X}}$ and $\varepsilon_{Z}(r)=\frac{e^{-r/\tau_{A}^{Z}}-e^{-r/\tau_{B}^{Z}}}{\tau_{A}^{Z}-\tau_{B}^{Z}}$, and synaptic delays in the feedforward excitatory and feedback inhibitory connections are *d*_*ij*_^*X*^ and *d*_*jk*_^*Z*^. For feedforward excitatory connections, the synaptic delay *d*_*ij*_^*X*^ is given by the sum of the axonal delay *d*_*ij*_^*a*^ and dendritic delay *d*_*ij*_^*d*^, whereas for inhibitory connections, we assume for simplicity that the delay is purely axonal. The response of the output neuron follows $y_{j}(t)=\hat{\sigma }\left[g_{E}(u_{j}^{E})\right]$. Similarly, inhibitory neurons in the lateral layer show Poisson firing based on the membrane potential {*u* ^*I*^_k_}_*k* = 1,…,*N*_ which is defined as

$$u_{k}^{I}(t)=\sum_{j=1}^{M}w_{kj}^{Y}\int_{0}^{\infty }\varepsilon_{Y}(r)y_{j}(t-r-d_{kj}^{Y})dr,$$ (16)

for EPSPs of a lateral connection *w* ^*Y*^_*kj*_, convolution function $\varepsilon_{Y}(r)=\frac{e^{-r/\tau_{A}^{Y}}-e^{-r/\tau_{B}^{Y}}}{\tau_{A}^{Y}-\tau_{B}^{Y}}$, and synaptic delay of the lateral connection *d* ^*Y*^_*kj*_. The synaptic delay of the excitatory lateral connection is also approximated as the axonal delay. The spiking activity of the inhibitory neurons is given with $z_{k}(t)=\hat{\sigma }\left[g_{I}(u_{k}^{I})\right]$. For analytical tractability, we use a linear response curve g_E_(*u*) = *u* and g_I_(*u*) = *u*.

#### Synaptic plasticity

For most of this study, we focused on synaptic plasticity in the feedforward connection *W*_*X*_, with fixed lateral synaptic weights *W*_*Y*_ and *W*_*Z*_. When the timing of the spikes at the cell bodies of pre- and postsynaptic neurons is *t*_*pre*_ and *t*_*post*_, spike timings at the synaptic sites are $t_{pre}^{s}=t_{pre}+d_{ji}^{a}$ and $t_{post}^{s}=t_{post}+d_{ji}^{d}$ with axonal and dendritic delays of *d* ^*a*^_*ji*_ and *d* ^*d*^_*ji*_. For every pair of *t* ^*s*^_*pre*_ and *t* ^*s*^_*post*_, synaptic weight change is given with

$$\Delta w_{ji}^{X}=\left\{ \begin{array}{l} \eta^{X}f_{p}(w_{ji}^{X})\exp\left[-\left(t_{post}^{s}-t_{pre}^{s}\right)/\tau_{p}\right]\text{ (if }t_{post}^{s}>t_{pre}^{s}\text{)}\\ \eta^{X}f_{d}(w_{ji}^{X})\exp\left[-\left(t_{pre}^{s}-t_{post}^{s}\right)/\tau_{d}\right]\text{ (if }t_{post}^{s}<t_{pre}^{s}\text{)} \end{array}\right..$$ (17)

For the synaptic weight dependence of STDP, we considered a pairwise log-STDP [31] in which LTP/LTD follows

$$f_{p}(w)=C_{p}(1+\sigma_{stdp}\xi )e^{-w/(\beta w_{o})},f_{d}(w)=-C_{d}(1+\sigma_{stdp}\xi )\frac{\log(1+\alpha w/w_{o})}{\log(1+\alpha )},$$ (18)

where ξ is a Gaussian random variable. The log-weight dependence well replicates experimentally observed synaptic weight distributions [32,33] and is suggested to have an important function in memory modulation [82]. Analytical treatment below is applicable to other types of synaptic weight dependence, yet in the additive STDP (i.e. *f*_*p*_(*w*) = *C*_*p*_ and *f*_*d*_(*w*) = *C*_*d*_), the mean-field equation typically does not have any stable fixed point. In addition, under the multiplicative STDP in which LTD has a linear rather than a logarithmic dependence on synaptic weight, strong correlation is often necessary to induce salient LTP [31]. The coefficients *C*_*p*_ = 1 and $C_{d}=C_{p}\tau_{p}^{X}/\tau_{d}^{X}$ are chosen so that total LTP and LTD are balanced around the referential synaptic weight.

The STDP at E-to-I connections and I-to-E connections is similarly defined. For simplicity, we assume that synaptic delays are solely axonal (i.e., $d_{k,j}^{Y}=d_{k,j}^{Y,a}$,$d_{k,j}^{Z}=d_{k,j}^{Z,a}$), and the change in synaptic weight does not depend on the synaptic weight. To maintain the balance between LTP and LTD, coefficients are chosen as $C_{p}^{Y}=1$,$C_{d}^{Y}=\gamma^{Y}C_{p}^{Y}\tau_{p}^{Y}/\tau_{d}^{Y}$ ,$\eta^{Y}=0.3\eta w_{o}^{Y}/w_{o}^{X}$. Similarly, for I-to-E connections, $C_{p}^{Z}=1$,$C_{d}^{Z}=\gamma^{Z}C_{p}^{Z}\tau_{p}^{Z}/\tau_{d}^{Z}$ ,$\eta^{Z}=0.3\eta w_{o}^{Z}/w_{o}^{X}$. We also modify constant (initial) synaptic weights to *w*_*o*_^*Y*^ = 50.0 and *w*_*o*_^*Z*^ = 25.0, and bounded synaptic weights with *w* ^*Y*^_*max*_ = 100.0 and *w* ^*Z*^_*max*_ = 50.0. In this normalization, the total lateral inhibition takes the same value as that in the non-plastic model at the initial state. Time windows are defined as *τ*_*p*_^*Y*^ = *τ*_*d*_^*Y*^ = *τ*_*p*_^*Z*^ = *τ*_*d*_^*Z*^ = 20.0 ms.

In Fig 6C, anti-Hebbian STDP was calculated by

$$\Delta w^{Q}=\left\{ \begin{array}{l} -\eta^{Q}\exp\left[-\left(t_{post}^{s}-t_{pre}^{s}\right)/\tau_{d}^{Q}\right]\text{ (if }t_{post}^{s}>t_{pre}^{s}\text{)}\\ \eta^{Q}\gamma^{Q}(\tau_{d}^{Q}/\tau_{p}^{Q})\exp\left[-\left(t_{pre}^{s}-t_{post}^{s}\right)/\tau_{p}^{Q}\right]\text{ (if }t_{post}^{s}<t_{pre}^{s}\text{)} \end{array}\right.$$

for Q = Y or Z. Similarly, the correlation detector type of STDP in S2 Fig was defined as

$$\Delta w^{Q}=\left\{ \begin{array}{l} \eta^{Q}\left(\exp\left[-\left(t_{post}^{s}-t_{pre}^{s}\right)/\tau_{p}^{Q}\right]-(\tau_{p}^{Q}/\tau_{d}^{Q})\exp\left[-\left(t_{post}^{s}-t_{pre}^{s}\right)/\tau_{d}^{Q}\right]\right)\text{ (if }t_{post}^{s}>t_{pre}^{s}\text{)}\\ \eta^{Q}\gamma^{Q}\left(\exp\left[-\left(t_{post}^{s}-t_{pre}^{s}\right)/\tau_{p}^{Q}\right]-(\tau_{p}^{Q}/\tau_{d}^{Q})\exp\left[-\left(t_{post}^{s}-t_{pre}^{s}\right)/\tau_{d}^{Q}\right]\right)\text{ (if }t_{post}^{s}<t_{pre}^{s}\text{)} \end{array}\right.$$

The anti-correlation detector was calculated by changing the sign of above equations.

#### Leaky Integrate-and-Fire (LIF) model

In the main text, we performed all simulations with a linear Poisson model for analytical purposes, although we also confirmed those results with a conductance-based LIF model (S1 Fig). In the LIF model, the membrane potentials of excitatory neurons follow

$$\begin{array}{l} \frac{dv_{j}^{E}}{dt}=-\frac{1}{\tau_{m}^{E}}\left(v_{j}^{E}-V_{L}\right)-g_{j}^{EE}\left(v_{j}^{E}-V_{E}\right)-g_{j}^{EI}\left(v_{j}^{E}-V_{I}\right),\\ \frac{dg_{j}^{EE}}{dt}=-\frac{g_{j}^{EE}}{\tau_{s}^{EE}}+\overset{L}{\underset{i=1}{\sum }}w_{ji}^{X}\underset{t_{i}^{s}}{\sum }\delta (t-t_{i}^{s}),and\frac{dg_{j}^{EI}}{dt}=-\frac{g_{j}^{EI}}{\tau_{s}^{EI}}+\overset{N}{\underset{k=1}{\sum }}w_{jk}^{Z}\underset{t_{k}^{s}}{\sum }\delta (t-t_{k}^{s}), \end{array}$$

where *g*_*j*_^*EE*^ and *g*_*j*_^*EI*^ are excitatory and inhibitory conductances, respectively, and *t*_*i*_^*s*^ and *t*_*k*_^*s*^ are the spike timings of input neuron *i* and lateral neuron *k*. Similarly, for inhibitory neurons in the lateral layer,

$$\begin{array}{l} \frac{dv_{k}^{I}}{dt}=-\frac{1}{\tau_{m}^{I}}\left(v_{k}^{I}-V_{L}\right)-g_{k}^{IE}\left(v_{k}^{I}-V_{E}\right)-g_{k}^{II}\left(v_{k}^{I}-V_{I}\right),\\ \frac{dg_{k}^{IE}}{dt}=-\frac{g_{k}^{IE}}{\tau_{s}^{IE}}+\overset{M}{\underset{j=1}{\sum }}w_{kj}^{Y}\underset{t_{j}^{s}}{\sum }\delta (t-t_{j}^{s}),\;and\frac{dg_{k}^{II}}{dt}=-\frac{g_{k}^{II}}{\tau_{s}^{II}}+w_{II}\underset{t_{r}^{s}}{\sum }\delta (t-t_{r}^{s}). \end{array}$$

In addition to the excitatory inputs from the output layer, we added random inhibitory inputs as Poisson processes with a fixed firing rate *r*_*o*_^*II*^ for inhibitory neurons. A neuron fires if the membrane potential exceeds the threshold *V*_*th*_, and immediately goes into a refractory period in which the membrane potential stays at *V*_*ref*_ for 1 ms after spiking. Plasticity was implemented for *w*_*ji*_^*X*^ in the same manner as that for the Poisson model. Parameters were chosen as *V*_*L*_ = -70.0, *V*_*E*_ = 0.0, *V*_*I*_ = -80.0, *V*_*ref*_ = -60.0, *V*_*th*_ = -50.0 mV, *t*_*m*_^*E*^ = 20.0, *t*_*m*_^*I*^ = 10.0, *tsEE* = 5.0, *t*_*s*_^*EI*^ = 2.5, *t*_*s*_^*IE*^ = 4.0, *t*_*s*_^*II*^ = 5.0 ms, *w*_*o*_^*X*^ = 0.001, *w*_*o*_^*I*^ = 0.008, *w*_*o*_^*L*^ = 1.0, *r*_*o*_^*II*^ = 1000.0 Hz, *w* ^*II*^_*o*_ = 0.005, *C*_*d*_ = 1.8*C*_*p*_ *τ*_*p*_^*X*^/*τ*_*d*_^*X*^, and α = 50.0. All other parameters were the same as those used in the Poisson model (Table 2).

In the LIF model, synaptic weights develop in a manner similar to that for the linear Poisson model, although change occurs more rapidly (Fig 1B, S1A Fig). Both cross-correlation and mutual information behave as they do in the Poisson model, but the performance is slightly better, possibly because the dynamics are deterministic (Fig 1D and 1E, S1B and S1C Fig); however, membrane potentials show different responses for correlation events (S1D Fig) because output neurons are constantly in high-conductance states, so that correlation events immediately cause spikes. As a result, membrane potentials drop to the *V*_*ref*_, and the average potential goes down. Interestingly, after neuron groups detect different signals, a preferred signal initially causes hyperpolarization due to firing, but, subsequently, a non-preferred signal causes hyperpolarization due to lateral inhibition (Fig 1D right). The PSTH of firing shows that the behavior of the membrane potential in the Poisson model is similar (Fig 1C and S1E Fig). This is natural, because in the linear Poisson model, the firing rate has linear relationship with the membrane potential, whereas in LIF model relationship between the average membrane potential and firing rate is highly non-linear.

#### Bayesian ICA

If discretized with Δt, the time series of the external source activity is written as $S={\left\{s_{\mu k}\right\}}_{\mu =1,...,p}^{k=1,...,T/\Delta t}$, and input activity becomes $X={\left\{x_{ik}\right\}}_{i=1,..,L}^{k=1,...,T/\Delta t}$. Therefore, for prior information *I*, the joint probability of sources S and the estimated response probability matrix Q is

$$P\left[S,\tilde{Q}|X,I\right]=P\left[X|S,\tilde{Q},I\right]P\left[S,\tilde{Q}|I\right]/P\left[X|I\right].$$

Therefore, by considering marginal probability,

$$P\left[\tilde{Q}|X,I\right]=\frac{P\left[\tilde{Q}|I\right]}{P\left[X|I\right]}\int P\left[X|S,\tilde{Q},I\right]P\left[S|I\right]dS.$$ (19)

By considering maximum likelihood estimation for a given prior P[*S*|*I*], Q can be optimally estimated [46,47]. In our problem setting, by assuming that external signals are independent, and input neurons respond to signals with a Bernoulli process,

$$P\left[X|S,\tilde{Q},I\right]=\prod_{k=1}^{T/\Delta t}\prod_{i=1}^{L}\left[x_{i}^{k}p_{i}^{k}+(1-x_{i}^{k})(1-p_{i}^{k})\right],P\left[S|I\right]=\prod_{k=1}^{T/\Delta t}\prod_{i=1}^{L}{\left(r_{s}\Delta t\right)}^{s_{\mu }^{k}}{\left(1-r_{s}\Delta t\right)}^{1-s_{\mu }^{k}},$$

where

$$p_{i}^{k}=1-\left(1-r_{i}^{o}\Delta t\right)\prod_{\mu =1}^{p}\left[1-{\tilde{q}}_{i\mu }\sum_{k^{\prime }=0}^{\infty }\phi_{k^{\prime }}s_{\mu }^{k-k^{\prime }}\right],\phi_{k}=\frac{1}{2\theta_{t}{}^{3}}{\left[\left(k+1/2\right)\Delta t\right]}^{2}\exp\left[-\left(k+1/2\right)\Delta t/\theta_{t}\right].$$

Therefore, log-likelihood becomes

$$\log P\left[\tilde{Q}|X,I\right]=\log\left(\int dS\prod_{k=1}^{T/\Delta t}\left[\prod_{i=1}^{L}\left(x_{i}^{k}p_{i}^{k}+(1-x_{i}^{k})(1-p_{i}^{k})\right)\times \prod_{\mu =1}^{p}{\left(r_{s}\Delta t\right)}^{s_{\mu }^{k}}{\left(1-r_{s}\Delta t\right)}^{1-s_{\mu }^{k}}\right]\right).$$ (20)

By taking gradient descendent,

$$\frac{\partial }{\partial {\tilde{q}}_{i\mu }}\log P\left[\tilde{Q}|X,I\right]=\frac{1}{Z_{p}}\sum_{k=1}^{T/\Delta t}\int P[S,X|\tilde{Q},I]\frac{2x_{i}^{k}-1}{x_{i}^{k}p_{i}^{k}/\left(1-p_{i}^{k}\right)+(1-x_{i}^{k})}\frac{\sum_{k^{\prime }=0}^{\infty }\phi_{k^{\prime }}s_{\mu }^{k-k^{\prime }}}{1-{\tilde{q}}_{i\mu }\sum_{k^{\prime }=0}^{\infty }\phi_{k^{\prime }}s_{\mu }^{k-k^{\prime }}}dS.$$

Therefore, we need to calculate the integral over all possible combinations of sources in the past to obtain stochastic gradient descendent; however, such a calculation is computationally difficult and incompatible with neural computation. Instead, we used sequential sampling of $Y={\left\{y_{\mu k}\right\}}_{\mu =1,...,p}^{k=1,...,T/\Delta t}$, which is randomly sampled from

$$\begin{array}{l} P\left[y^{k}=s^{k}\right]\propto P\left[s^{k},x^{k}|Y^{1:k-1},\tilde{Q},I\right]\\ \;=\prod_{i=1}^{L}\left(x_{i}^{k}p_{i}^{k}(s^{k},Y^{1:k-1})+(1-x_{i}^{k})\left(1-p_{i}^{k}(s^{k},Y^{1:k-1})\right)\right)\times \prod_{\mu =1}^{p}{\left(r_{s}\Delta t\right)}^{y_{\mu }^{k}}{\left(1-r_{s}\Delta t\right)}^{1-y_{\mu }^{k}}, \end{array}$$ (21)

where

$$p_{i}^{k}(y^{k},Y^{1:k-1})=1-\left(1-r_{i}^{o}\Delta t\right)\prod_{\mu =1}^{p}\left[1-{\tilde{q}}_{i\mu }\sum_{k^{\prime }=0}^{\infty }\phi_{k^{\prime }}y_{\mu }^{k-k^{\prime }}\right].$$

Note in the above equations, *x* ^*k*^ is given as a fixed value and not a random variable. Under this sample-based approximation, the stochastic gradient descendent follows

$$\Delta {\tilde{q}}_{i\mu }^{k}\propto \frac{2x_{i}^{k}-1}{x_{i}^{k}p_{i}^{k}(y^{k},Y^{1:k-1})/\left(1-p_{i}^{k}(y^{k},Y^{1:k-1})\right)+(1-x_{i}^{k})}\times \frac{\sum_{k^{\prime }=0}^{\infty }\phi_{k^{\prime }}y_{\mu }^{k-k^{\prime }}}{1-{\tilde{q}}_{i\mu }\sum_{k^{\prime }=0}^{\infty }\phi_{k^{\prime }}y_{\mu }^{k-k^{\prime }}}.$$ (22)

For Fig 7C, we discretized the activity of hidden sources and input neurons with 5 ms bins, and performed learning with a learning rate *η* ^*SGD*^ = 0.001. Cross-correlation was evaluated using the sample sequence Y. For the ideal case, we performed sequential sampling from the true response probability Q.

If y^k-k’^_μ_ = 1 and y^k-k”^_μ_ = 0 for all other nearby k” (≠k’), and if *q*_*iν*_ = 0 for all ν (≠μ), then LTP at the connection *q*_*iμ*_ caused by an output spike y^k-k’^_*μ*_ = 1 for x_i_^k^ = 1 is written as

$$\Delta q_{i\mu }^{k,k^{\prime },LTP}=\frac{\left(1-\left[r_{o}^{X}-r_{o}^{S}{\tilde{q}}_{i\mu }\right]\right)\left(1-{\tilde{q}}_{i\mu }\phi_{k^{\prime }}\right)}{1-\left(1-\left[r_{o}^{X}-r_{o}^{S}{\tilde{q}}_{i\mu }\right]\right)\left(1-{\tilde{q}}_{i\mu }\phi_{k^{\prime }}\right)}\times \frac{\phi_{k^{\prime }}}{1-{\tilde{q}}_{i\mu }\phi_{k^{\prime }}}.$$ (23)

In the absence of the input spike (x_i_^k^ = 0), an output spike y^k-k’^_*μ*_ = 1 causes LTD in total $\Delta q_{i\mu }^{LTD}=-\sum_{k^{\prime }=0}^{\infty }\frac{\phi_{k^{\prime }}}{1-{\tilde{q}}_{i\mu }\phi_{k^{\prime }}}$. Therefore, this learning rule has weight dependence and temporal dependence similar to those in STDP. In Fig 7D, we plotted $\Delta q_{i\mu }^{k,k^{\prime },LTP}$ and $\Delta q_{i\mu }^{LTD}$ for different ${\tilde{q}}_{i\mu }$ (${\tilde{q}}_{i\mu }$= 0.1, 0.3, 0.5).

#### Blind source separation

In the blind source separation task, we created the original source by calculating high-frequency and low-frequency components separately. First, the spectrum of the signal *q* at a high frequency was defined as

$$a_{h}^{q}(f)=\sum_{i}\sum_{k}\frac{a_{h,i}^{q}b_{h,k}^{q}}{\sqrt{2\pi }\sigma_{hf}}\exp\left[-{\left(f-kf_{h,i}^{q}\right)}^{2}/\left(2\sigma_{hf}{}^{2}\right)\right],$$

where *f* ^*q*^_*h*,*i*_ is a characteristic frequency of signal *q*, and *kf* ^*q*^_*h*,*i*_ are the harmonics of that frequency. The standard deviation was defined as $\sigma_{h,f}=k\sigma_{h,f}^{o}$ for $\sigma_{h,f}^{o}=20Hz$. Low-frequency components were directly given as an exponential oscillation as below.

$$a_{l}^{q}(t)=\frac{1}{Z_{l}}\exp\left[\beta_{l}\sum_{i}a_{l,i}^{q}\cos\left(2\pi f_{l,i}^{q}\left(t-\delta_{l,i}^{q}\right)\right)\right],$$

*f* ^*q*^_*l*,*i*_ is a characteristic frequency, and *δ* ^*q*^_*l*,*i*_ is the delay. By combining these two components, the amplitude of a mixed sound is given as

$$a(t)=\sum_{q}a_{l}^{q}(t)\sum_{i}a_{h}^{q}(f_{i})\cos\left(2\pi f_{i}\left(t-\delta_{f}^{q}\right)\right).$$

Summation over frequency *f* is performed using 400 representative values that correspond to the tuned frequency of each input neuron:

$$f_{i}=\exp\left[\frac{i}{L}\left(\log f_{\max}-\log f_{\min}\right)+\log f_{\min}\right].$$

In neural implementation, input neurons were stimulated with the response probability $q_{i}(t)=q_{o}\sum_{q}a_{l}^{q}(t)a_{h}^{q}(f_{i})$ where *q*_*o*_ = 0.05.

In the simulated example, for high-frequency components, we defined *f* ^*q*^_*h*,*I*_ = {{523.3,784.0}, {587.4,880.0}, {650.0,830.6}, {698.5,932.4}}, *a* ^*q*^_*h*,*I*_ = {{0.6,0.4}, {0.3,0.7}, {0.5,0.5}, {0.9,0.3}}, *b* ^*q*^_*h*,*k*_ = {{1.0,0.5,0.2,0.1}, {1.0,0.5,0.3,0.2}, {1.0,0.1,1.0,0.8}, {1.0,0.8,0.1,0.1}}, and *σ* ^*o*^_*h*,*f*_ = 20 Hz. Each column represents four different sources. Similarly for low-frequency components, we used *f* ^*q*^_*l*,*I*_ = {{0.4,5.0,10.0,40.0,88.0}, {0.6,6.0,8.0,42.0,86.0}, {0.2,4.0,7.5,44.0,84.0}, {0.3,6.0,7.0,46.0,82.0}}, *a* ^*q*^_*l*,*I*_ = {{0.3,0.4,0.2,0.5,0.5}, {0.25,0.5,0.2,0.5,0.5}, {0.24,0.3,0.4,0.5,0.5}, {0.61,0.2,0.2,0.5,0.5}}, *δ* ^*q*^_*l*,*I*_ = {{1.0,0.25,0.65,0.17,0.01}, {3.0,0.12,0.32,0.13,0.02}, {7.8,0.55,0.40,0.11,0.03}, {4.5,0.22,0.71,0.07,0.05}}, β_*l*_ = 5.0, and *Z*_*l*_ = 27.24. We chose *f*_*min*_ = 500 Hz, *f*_*min*_ = 4,500 Hz, and *δ* ^*q*^_*f*_ was randomly selected from 0 to 1/*f*_*min*_. Fig 8A was generated by performing Fourier transformations with 25 ms sliding bins at every 2.5 ms.

#### Details of the simulation

Simulations were calculated using the Runge-Kutta method, with a 0.05 ms time step. Initial synaptic weights were randomly chosen with $w_{ij}^{Q}=w_{o}^{Q}(1+\sigma_{W}^{init}\zeta )$ for Q = X, Y, Z and a random Gaussian variable ξ. Similarly, synaptic delays were decided as $d_{ij}^{Q}=d_{\min}^{Q}+(d_{\min}^{Q}-d_{\max}^{Q})\xi$ for a random variable ξ uniformly chosen from [0,1].

### Analytical consideration of synaptic weight dynamics

#### Correlation among input neurons

Because input neurons receive common inputs from external sources, we define cross-correlation among input neurons as *C*_*il*_(s) ≡ 〈*x*_*i*_(*t*)*x*_*l*_(t-s)〉-〈*x*_*i*_(*t*)〉〈*x*_*l*_(*t*)〉, and cross-correlation among input neurons satisfies

$$\begin{array}{l} C_{il}(s)=\langle \hat{\sigma }\left[r_{i}^{o}+\sum_{\mu =1}^{p}q_{i\mu }\int_{0}^{\infty }\phi (t^{\prime })s_{\mu }(t-t^{\prime })dt^{\prime }\right]\times \hat{\sigma }\left[r_{l}^{o}+\sum_{\mu =1}^{p}q_{l\mu }\int_{0}^{\infty }\phi (t^{\prime \prime })s_{\mu }(t-s-t^{\prime \prime })dt^{\prime \prime }\right]\rangle -{\left(\nu_{o}^{X}\right)}^{2}\\ ≅\nu_{o}^{S}\sum_{\mu =1}^{p}q_{i\mu }q_{l\mu }\int_{0}^{\infty }dt^{\prime }\int_{0}^{\infty }dt^{\prime \prime }\phi (t^{\prime })\phi (t^{\prime \prime })\delta (t^{\prime }-t^{\prime \prime }-s)=\nu_{o}^{S}\sum_{\mu =1}^{p}q_{i\mu }q_{l\mu }\int_{\max(0,s)}^{\infty }dt^{\prime }\phi (t^{\prime })\phi (t^{\prime }-s). \end{array}$$ (24)

When $\phi (t)=t^{2}e^{-t/\theta_{t}}/2\theta_{t}{}^{3}$, *C*_*il*_ *(s)* becomes

$$C_{il}(s)=\nu_{o}^{S}\sum_{\mu =1}^{p}q_{i\mu }q_{l\mu }\frac{1}{16\theta_{t}{}^{3}}\left(s^{2}+3\theta_{t}\left|s\right|+3\theta_{t}{}^{2}\right)e^{-\left|s\right|/\theta_{t}}=\nu_{o}^{S}\sum_{\mu =1}^{p}q_{i\mu }q_{l\mu }h(s),$$

where $h(s)\equiv \frac{1}{16\theta_{t}{}^{3}}\left(s^{2}+3\theta_{t}\left|s\right|+3\theta_{t}{}^{2}\right)e^{-\left|s\right|/\theta_{t}}.$

#### Average synaptic weight velocity

The synaptic weight dynamics defined above can be rewritten as

$$\frac{dw_{ji}^{X}}{dt}=x_{i}(t-d_{ji}^{Xa})\int_{0}^{\infty }F_{d}(w_{ji}^{X},s)y_{j}(t-s-d_{ji}^{Xd})ds+y_{j}(t-d_{ji}^{Xd})\int_{0}^{\infty }F_{p}(w_{ji}^{X},s)x_{i}(t-s-d_{ji}^{Xa})ds$$ (25)

for $F_{d}(w_{ij}^{X},s)=f_{d}(w_{ij}^{X})e^{-s/\tau_{{}_{d}}}$,$F_{p}(w_{ij}^{X},s)=f_{p}(w_{ij}^{X})e^{-s/\tau_{{}_{p}}}$. By taking an average over a short period of time and also using a stochastic Poisson process, synaptic weight change follows

$$\begin{array}{l} \langle \frac{dw_{ji}^{X}}{dt}\rangle =\langle x_{i}(t-d_{ji}^{Xa})\int_{0}^{\infty }F_{d}(w_{ji}^{X},s)y_{j}(t-s-d_{ji}^{Xd})ds\rangle +\langle y_{j}(t-d_{ji}^{Xd})\int_{0}^{\infty }F_{p}(w_{ji}^{X},s)x_{i}(t-s-d_{ji}^{Xa})ds\rangle \\ =\langle x_{i}(t-d_{ji}^{Xa})\int_{-\infty }^{0}F_{d}(w_{ji}^{X},-s^{\prime })y_{j}(t+s^{\prime }-d_{ji}^{Xd})ds^{\prime }\rangle +\langle y_{j}(t-d_{ji}^{Xd})\int_{0}^{\infty }F_{p}(w_{ji}^{X},s)x_{i}(t-s-d_{ji}^{Xa})ds\rangle \\ =\langle \int_{-\infty }^{0}F_{d}(w_{ji}^{X},-s^{\prime })x_{i}(t^{\prime }-s^{\prime }-d_{ji}^{Xa})y_{j}(t^{\prime }-d_{ji}^{Xd})ds^{\prime }\rangle +\langle \int_{0}^{\infty }F_{p}(w_{ji}^{X},s)x_{i}(t-s-d_{ji}^{Xa})y_{j}(t-d_{ji}^{Xd})ds\rangle \\ ≅\int_{-\infty }^{\infty }F(w_{ji}^{X},s)\langle x_{i}(t-s-d_{ji}^{Xa})y_{j}(t-d_{ji}^{Xd})\rangle ds, \end{array}$$

where $F(w,s)\equiv \left\{ \begin{array}{l} F_{p}(w,s)\text{ if( }s\geq 0\text{ )}\\ F_{d}(w,-s)\text{ if( }s<0\text{ )} \end{array}\right..$

Therefore, by calculating the cross-correlation between pre-spikes *x*_*i*_ and post-spikes *y*_*j*_, synaptic weight dynamics can be analytically estimated. Because the spike probability linearly depends on the membrane potential in our model, cross-correlation follows

$$\begin{array}{l} \langle x_{i}(t-s-d_{ji}^{Xa})y_{j}(t-d_{ji}^{Xd})\rangle ≅\langle x_{i}(t-s-d_{ji}^{Xa})u_{j}^{E}(t-d_{ji}^{Xd})\rangle \\ ≅\sum_{l=1}^{L}w_{jl}^{X}\int_{0}^{\infty }dr\varepsilon_{X}(r)\langle x_{i}(t-s-d_{ji}^{Xa})x_{l}(t-d_{ji}^{Xd}-r-d_{ji}^{X})\rangle -\sum_{k=1}^{N}w_{jk}^{Z}\int_{0}^{\infty }dr\varepsilon_{Z}(r)\langle x_{i}(t-s-d_{ji}^{Xa})z_{k}(t-d_{ji}^{Xd}-r-d_{jk}^{Z})\rangle . \end{array}$$

Since we define cross-correlation among input neurons as

$$C_{il}(s)\equiv \langle x_{i}(t)x_{l}(t-s)\rangle -\langle x_{i}(t)\rangle \langle x_{l}(t)\rangle ,$$

the first term is written as

$$\sum_{l=1}^{L}w_{jl}^{X}\int_{0}^{\infty }dr\varepsilon_{X}(r)\langle x_{i}(t-s-d_{ji}^{Xa})x_{l}(t-d_{ji}^{Xd}-r-d_{ji}^{X})\rangle ≅\sum_{l=1}^{L}w_{jl}^{X}\left[{\left(\nu_{o}^{X}\right)}^{2}+\int_{0}^{\infty }dr\varepsilon_{X}(r)C_{il}(r-s+2d_{Xd})\right].$$ (26)

This result is consistent with that in previous studies [5,38,45]. The analysis can be extended to the cross-correlation between an input neuron and a lateral inhibitory neuron as

$$\begin{array}{l} \langle x_{i}(t-s-d_{ji}^{Xa})z_{k}(t-d_{ji}^{Xd}-r-d_{jk}^{Z})\rangle ≅\langle x_{i}(t-s-d_{ji}^{Xa})u_{k}^{I}(t-d_{ji}^{Xd}-r-d_{jk}^{Z})\rangle \\ ≅\sum_{m=1}^{M}w_{km}^{Y}\int_{0}^{\infty }dq\varepsilon_{Y}(q)\langle x_{i}(t-s-d_{ji}^{Xa})y_{m}(t-d_{ji}^{Xd}-r-d_{jk}^{Z}-q-d_{km}^{Y})\rangle \\ ≅\sum_{m=1}^{M}w_{km}^{Y}\sum_{l=1}^{L}w_{ml}^{X}\int_{0}^{\infty }dq\varepsilon_{Y}(q)\int_{0}^{\infty }dr^{\prime }\varepsilon_{X}(r^{\prime })\langle x_{i}(t-s-d_{ji}^{Xa})x_{l}(t-d_{ji}^{Xd}-r-d_{jk}^{Z}-q-d_{km}^{Y}-r^{\prime }-d_{ml}^{X})\rangle \\ -\sum_{m=1}^{M}w_{km}^{Y}\sum_{n=1}^{N}w_{mn}^{Z}\int_{0}^{\infty }dq\varepsilon_{Y}(q)\int_{0}^{\infty }dr^{\prime }\varepsilon_{Z}(r^{\prime })\langle x_{i}(t-s-d_{ji}^{Xa})z_{n}(t-d_{ji}^{Xd}-r-d_{jk}^{Z}-q-d_{km}^{Y}-r^{\prime }-d_{mn}^{Z})\rangle \\ ≅\sum_{m=1}^{M}w_{km}^{Y}\sum_{l=1}^{L}w_{ml}^{X}\left[{\left(\nu_{o}^{X}\right)}^{2}+\int_{0}^{\infty }dq\varepsilon_{Y}(q)\int_{0}^{\infty }dr^{\prime }\varepsilon_{X}(r^{\prime })C_{il}(r+q+r^{\prime }-s+2d_{Xd}+d_{Z}+d_{Y})\right]-\sum_{m=1}^{M}w_{km}^{Y}\sum_{n=1}^{N}w_{mn}^{Z}\nu_{o}\nu_{n}^{Z}. \end{array}$$ (27)

Theoretically, expansion over a lateral connection should be performed infinite times to obtain the exact solution, but at each expansion, the delay caused by synaptic delay *d*_*Z*_+*d*_*Y*_ and EPSP/IPSP rise times is accumulated so that the effect on correlation rapidly becomes small, especially when the original input cross-correlation C(t) is narrow; however, even if C(t) is broad, the effect for learning is bounded by the STDP time window. Therefore, higher order terms practically influence weight dynamics only through firing rates, so that by applying the approximation

$$\int_{0}^{\infty }dq\varepsilon_{Y}(q)\int_{0}^{\infty }dr^{\prime }\varepsilon_{Z}(r^{\prime })\langle x_{i}(t-s-d_{ji}^{Xa})z_{n}(t-d_{ji}^{Xd}-r-d_{jk}^{Z}-q-d_{km}^{Y}-r^{\prime }-d_{mn}^{Z})\rangle ≅\nu_{o}^{X}\nu_{n}^{Z},$$

the last term can be obtained. In general, *ν*_*n*_^*Z*^ is not analytically calculable, but by considering the balanced condition, it can be estimated. Therefore, the second term is given as

$$\begin{array}{l} \sum_{k=1}^{N}w_{jk}^{Z}\int_{0}^{\infty }dr\varepsilon_{Z}(r)\langle x_{i}(t-s-d_{ji}^{Xa})z_{k}(t-d_{ji}^{Xd}-r-d_{jk}^{Z})\rangle \\ ≅\sum_{k=1}^{N}w_{jk}^{Z}\sum_{m=1}^{M}w_{km}^{Y}\sum_{l=1}^{L}w_{ml}^{X}\left[{\left(\nu_{o}^{X}\right)}^{2}+\int_{0}^{\infty }dr\varepsilon_{Z}(r)\int_{0}^{\infty }dq\varepsilon_{Y}(q)\int_{0}^{\infty }dr^{\prime }\varepsilon_{X}(r^{\prime })C_{il}(r+q+r^{\prime }-s+2d_{Xd}+d_{Z}+d_{Y})\right]\\ \;-\sum_{k=1}^{N}w_{jk}^{Z}\sum_{m=1}^{M}w_{km}^{Y}\sum_{n=1}^{N}w_{mn}^{Z}\nu_{o}^{X}\nu_{n}^{Z} \end{array}$$

Therefore, if we denote

$$\begin{array}{l} \Gamma_{il}^{X1}(w_{ji}^{X})\equiv \int_{-\infty }^{\infty }dsF(w_{ji}^{X},s)\int_{0}^{\infty }dr\varepsilon_{X}(r)C_{il}(r-s+2d_{Xd})\\ \Gamma_{il}^{X2}(w_{ji}^{X})\equiv \int_{-\infty }^{\infty }dsF(w_{ji}^{X},s)\int_{0}^{\infty }dr\varepsilon_{Z}(r)\int_{0}^{\infty }dq\varepsilon_{Y}(q)\int_{0}^{\infty }dr^{\prime }\varepsilon_{X}(r^{\prime })C_{il}(r+q+r^{\prime }-s+2d_{Xd}+d_{Z}+d_{Y})\\ \bar{F}(w_{ji}^{X})\equiv \int_{-\infty }^{\infty }F(w_{ji}^{X},s)ds, \end{array}$$ (28)

average synaptic weight dynamics satisfy

$$\begin{array}{l} \langle \frac{dw_{ji}^{X}}{dt}\rangle ≅\sum_{l=1}^{L}w_{jl}^{X}\Gamma_{il}^{X1}(w_{ji}^{X})-\sum_{k=1}^{N}w_{jk}^{Z}\sum_{m=1}^{M}w_{km}^{Y}\sum_{l=1}^{L}w_{ml}^{X}\Gamma_{il}^{X2}(w_{ji}^{X})\\ \;+\bar{F}(w_{ji}^{X})\left[\sum_{l=1}^{L}w_{jl}^{X}{\left(\nu_{o}^{X}\right)}^{2}-\sum_{k=1}^{N}w_{jk}^{Z}\sum_{m=1}^{M}w_{km}^{Y}\sum_{l=1}^{L}w_{ml}^{X}{\left(\nu_{o}^{X}\right)}^{2}+\sum_{k=1}^{N}w_{jk}^{Z}\sum_{m=1}^{M}w_{km}^{Y}\sum_{n=1}^{N}w_{mn}^{X}\nu_{o}^{X}\nu_{n}^{Z}\right]. \end{array}$$ (29)

The first two terms are Hebbian terms that depend on correlation by Γ^X1^ and Γ^X2^, whereas the remainders are homeostatic terms. In all terms, synaptic weight dependence is primarily caused by *w* ^*X*^_*ji*_ and not by other synapses.

By inserting the explicit representation of correlation into the equation above, Γ^X1^ and Γ^X2^ can be rewritten as

$$\begin{array}{l} \Gamma_{il}^{X1}(w_{ji}^{X})=\nu_{o}^{S}G_{1}^{X}(w_{ji}^{X})\sum_{\mu =1}^{p}q_{i\mu }q_{l\mu },\Gamma_{il}^{X2}(w_{ji}^{X})=\nu_{o}^{S}G_{2}^{X}(w_{ji}^{X})\sum_{\mu =1}^{p}q_{i\mu }q_{l\mu },\\ G_{1}^{X}(w_{ji}^{X})\equiv \int_{-\infty }^{\infty }dsF(w_{ji}^{X},s)\int_{0}^{\infty }dr\varepsilon_{X}(r)\int_{\max(0,r-s+2d_{Xd})}^{\infty }dt^{\prime }\phi (t^{\prime })\phi (t^{\prime }-(r-s+2d_{Xd})),\\ G_{2}^{X}(w_{ji}^{X})\equiv \int_{-\infty }^{\infty }dsF(w_{ji}^{X},s)\int_{0}^{\infty }dr\varepsilon_{Z}(r)\int_{0}^{\infty }dq\varepsilon_{Y}(q)\int_{0}^{\infty }dr^{\prime }\varepsilon_{X}(r^{\prime })\int_{\max(0,t^{\prime \prime })}^{\infty }dt^{\prime }\phi (t^{\prime })\phi (t^{\prime }-(r+q+r^{\prime }-s+2d_{Xd}+d_{Z}+d_{Y})) \end{array}$$ (30)

where *t*″ = *r*+*q*+*r*′-*s*+2*d*_*xd*_+*d*_*z*_+*d*_*Y*_. Note that *G*_*1*_^*X*^ and *G*_*2*_^*X*^ do not depend on any indexes of the neurons, except for synaptic weight dependency, and so the two values are considered basic constants that decide how correlation shapes learning.

If we ignore the homeostatic term, then the synaptic weight dynamic is written in the matrix form as $\overset{•}{W_{X}}\approx \left(W_{X}C^{t}\right).G_{1}^{X}-\left(W_{X}W_{Z}W_{Y}C^{t}\right).G_{2}^{X}$, where the dot product is defined as (*A*.*B*)_*ij*_ = *A*_*ij*_ *B*_*ij*_. Especially if we approximate *G*_*1*_^*X*^ and *G*_*2*_^*X*^ with *g*_*1*_^X^ ≡ *G*_*1*_^*X*^(*w*_*o*_^*X*^) and *g*_*2*_^X^ ≡ *G*_*2*_^*X*^(*w*_*o*_^*X*^) (or if weight dependence is negligible as in additive-STDP),$\overset{•}{W_{X}}\approx W_{X}\left(g_{1}^{X}E-g_{2}^{X}W_{Z}W_{Y}\right)C^{t}$.

The correlation kernel *χ*_*1*_^*X*^ was derived from

$$\begin{array}{c} G_{1}^{X}(w_{ji}^{X})=\int_{-\infty }^{\infty }dsF(w_{ji}^{X},s)\int_{0}^{\infty }dr\varepsilon_{X}(r)\int_{\max(0,r-s+2d_{Xd})}^{\infty }dt^{\prime }\int_{-\infty }^{\infty }d\tau \phi (t^{\prime })\phi (t^{\prime }-(r-s+2d_{Xd}))\delta \left(\tau -(r-s+2d_{Xd})\right)\\ =\int_{-\infty }^{\infty }d\tau \int_{-\tau +2d_{Xd}}^{\infty }dsF\left(w_{ji}^{X},s\right)\varepsilon_{X}\left(s-2d_{Xd}\right)\int_{\max\left(0,\tau \right)}^{\infty }dt^{\prime }\phi (t^{\prime })\phi (t^{\prime }-\tau )\\ =\int_{-\infty }^{\infty }\chi_{1}^{X}(\tau ;w_{ji}^{X})h(\tau )d\tau \end{array}$$ (31)

where $\chi_{1}^{X}\left(\tau ;w\right)=\int_{-\tau +2d_{Xd}}^{\infty }dsF\left(w,s\right)\varepsilon_{X}\left(\tau +s-2d_{Xd}\right)$, and $h(\tau ;\theta_{t})\equiv \int_{\max(\tau ,0)}^{\infty }dt^{\prime }\phi (t^{\prime })\phi (t^{\prime }-\tau )$. The second correlation kernel *χ*_*2*_^*X*^ was calculated in a similar way.

#### Mean-field approximation of a two-source model

If the correlation structure *C(s)* is simply organized, further analytical consideration is possible. In the two-source model shown in Fig 2A, lateral connections are structured non-reciprocally, and EPSP/IPSP sizes are constants. The synaptic weight matrices are written as

$$W_{km}^{Y}=\left\{ \begin{array}{l} w_{Y}\text{ (if }\left\lfloor k/N_{a}\right\rfloor \text{=}\left\lfloor m/M_{a}\right\rfloor \text{)}\\ 0\text{ (otherwise)} \end{array}\right.,W_{jk}^{Z}=\left\{ \begin{array}{l} w_{Z}\text{ (if }\left\lfloor j/M_{a}\right\rfloor \neq \left\lfloor k/N_{a}\right\rfloor \text{)}\\ 0\text{ (otherwise)} \end{array}\right..$$ (32)

Therefore, the original L × M differential equations can be reduced into 2 × 2 equations of representative neurons as

$$\begin{array}{l} \frac{dw_{\mu \nu }^{X}}{dt}≅\sum_{\nu^{\prime }=1}^{L/L_{a}}L_{a}w_{\mu \nu^{\prime }}^{X}\nu_{o}^{S}G^{X}(w_{\nu \nu^{\prime }}^{X})\sum_{\rho }q_{\nu \rho }q_{\nu^{\prime }\rho }-N_{a}w_{Z}M_{a}w_{Y}\sum_{\nu^{\prime }=1}^{L/L_{a}}L_{a}w_{\bar{\mu }\nu^{\prime }}^{X}\nu_{o}^{S}G^{Y}(w_{\nu \nu^{\prime }}^{X})\sum_{\rho }q_{\nu \rho }q_{\nu^{\prime }\rho }\\ \;+\bar{F}(w_{\mu \nu }^{X})\left[{\left(\nu_{o}^{X}\right)}^{2}\sum_{\nu^{\prime }=1}^{L/L_{a}}L_{a}w_{\mu \nu^{\prime }}^{X}-{\left(\nu_{o}^{X}\right)}^{2}N_{a}w_{Z}M_{a}w_{Y}\sum_{\nu^{\prime }=1}^{L/L_{a}}L_{a}w_{\bar{\mu }\nu^{\prime }}^{X}+{\left(N_{a}w_{Z}\right)}^{2}M_{a}w_{Y}\nu_{o}^{X}\nu_{\mu }^{Z}\right]. \end{array}$$ (33)

The firing rates of inhibitory neurons can be approximated as

$$\nu_{\mu }^{Z}≅\frac{1}{N_{a}}\sum_{k\in \Omega_{\mu }^{Z}}u_{k}^{I}≅M_{a}w_{Y}\nu_{\mu }^{Y}≅M_{a}w_{Y}\left(\left(L_{a}w_{\mu A}+L_{a}w_{\mu B}+2L_{a}w_{o}^{X}\right)\nu_{o}^{X}-N_{a}w_{z}\nu_{\bar{\mu }}^{Z}\right).$$ (34)

Therefore, by solving the simultaneous equations for ν_1_^Z^ and ν_2_^Z^,

$$\begin{array}{l} \nu_{1}^{Z}=\frac{M_{a}w_{Y}\nu_{o}^{X}}{1-{\left(M_{a}w_{Y}N_{a}w_{Z}\right)}^{2}}\left[\left(L_{a}w_{1A}+L_{a}w_{1B}+2L_{a}w_{o}^{X}\right)-\left(M_{a}w_{Y}N_{a}w_{Z}\right)\left(L_{a}w_{2A}+L_{a}w_{2B}+2L_{a}w_{o}^{X}\right)\right]\\ \nu_{2}^{Z}=\frac{M_{a}w_{Y}\nu_{o}^{X}}{1-{\left(M_{a}w_{Y}N_{a}w_{Z}\right)}^{2}}\left[\left(L_{a}w_{2A}+L_{a}w_{2B}+2L_{a}w_{o}^{X}\right)-\left(M_{a}w_{Y}N_{a}w_{Z}\right)\left(L_{a}w_{1A}+L_{a}w_{1B}+2L_{a}w_{o}^{X}\right)\right] \end{array}$$

This analytical approach is applicable only when the synaptic weight change is sufficiently slow relative to the neural dynamics. Also, because we ignored the variance in the synaptic weights, numerically the accuracy is limited.

#### Analytic approach for STDP in lateral and inhibitory connections

Using a similar calculation as above, synaptic weight development of the lateral connections is given as

$$\overset{•}{W_{Y}}\approx g_{1}^{Y}W_{Y}W_{X}C^{t}W_{X}^{t}-g_{2}^{Y}W_{Y}W_{X}C^{t}W_{X}^{t}W_{Y}^{t}W_{z}^{t}-g_{3}^{Y}W_{Y}W_{Z}W_{Y}W_{X}C^{t}W_{X}^{t},$$ (35)

where

$$\begin{array}{l} g_{1}^{Y}\equiv \int_{-\infty }^{\infty }dsF^{Y}(s)\int^{\text{​}}D_{r}^{X}\int^{\text{​}}D_{u}^{Y}\int^{\text{​}}D_{r^{\prime }}^{X}h(u+r^{\prime }-s-r)\\ g_{2}^{Y}\equiv \int_{-\infty }^{\infty }dsF^{Y}(s)\int D_{r}^{X}\int D_{u}^{Y}\int D_{q}^{Z}\int D_{u^{\prime }}^{Y}\int D_{r^{\prime }}^{X}h(u^{\prime }+r^{\prime }-s-q-u-r-d_{Y}-d_{Z})\\ g_{3}^{Y}\equiv \int_{-\infty }^{\infty }dsF^{Y}(s)\int D_{r}^{X}\int D_{u}^{Y}\int D_{q}^{Z}\int D_{u^{\prime }}^{Y}\int D_{r^{\prime }}^{X}h(u+q+u^{\prime }+r^{\prime }+d_{Y}+d_{Z}-s-r), \end{array}$$

where $\int D_{r}^{X}\equiv \int_{0}^{\infty }dr\varepsilon_{X}(r)$. The meaning of these equations is made clear by summarizing the correlation propagation in the diagrams (S2D i–iii Fig). In the diagram, blue wavy lines represent intrinsic correlation, and arrows are synaptic connections. To estimate how a blue correlation influences STDP at a red arrow, we need to determine all the major trajectories in which the correlation reaches pre- and postsynaptic neurons. In the linear Poisson framework, for a given trajectory, the propagation of a correlation is calculated by simply using integrals as above. From this diagram, we can safely assume that *g* ^*Y*^_*2*_ and *g* ^*Y*^_*3*_ are negligibly smaller than *g* ^*Y*^_*1*_, because trajectories (ii) and (iii) are secondary correlations and also contain synaptic delays. In this approximation, we additionally assume that

$$C=\left(\begin{array}{cc} c_{s} & 0\\ 0 & c_{s} \end{array}\right),W_{X}=\left(\begin{array}{cc} w_{s}^{X} & w_{w}^{X}\\ w_{w}^{X} & w_{s}^{X} \end{array}\right).$$

Then,

$$\begin{array}{l} \frac{d}{dt}\left(\begin{array}{c} w_{11}^{Y}\\ w_{12}^{Y}\\ w_{21}^{Y}\\ w_{22}^{Y} \end{array}\right)\approx \left(\begin{array}{cccc} A^{L} & B^{L} & 0 & 0\\ B^{L} & A^{L} & 0 & 0\\ 0 & 0 & A^{L} & B^{L}\\ 0 & 0 & B^{L} & A^{L} \end{array}\right)\left(\begin{array}{c} w_{11}^{Y}\\ w_{12}^{Y}\\ w_{21}^{Y}\\ w_{22}^{Y} \end{array}\right)\\ A^{L}\equiv c_{S}g_{1}^{Y}\left({\left(w_{s}^{X}\right)}^{2}+{\left(w_{w}^{X}\right)}^{2}\right),B^{L}\equiv 2c_{s}g_{1}^{Y}w_{s}^{X}w_{w}^{X} \end{array}$$

Therefore, $\left(w_{11}^{Y},w_{12}^{Y},w_{21}^{Y},w_{22}^{Y}\right)\propto \left(+1,-1,-1,+1\right)$ is a eigenvector of the transition matrix, and the eigenvalue is $c_{s}g_{1}^{Y}{\left(w_{s}^{X}-w_{w}^{X}\right)}^{2}$. Because the eigenvector develops by $\exp\left[c_{s}g_{1}^{Y}{\left(w_{s}^{X}-w_{w}^{X}\right)}^{2}t\right]$, when *g* ^*Y*^_*1*_ is positive, the E-to-I connections are more likely to be structured in a way that the inhibitory neurons become feature selective. On the other hand, if that value is negative, such structure may not be obtained. Note that (1, -1, -1, 1) is not the principal eigenvector in this simple analysis, because the eigensystem of the matrix is {{A^L^+B^L^, A^L^+B^L^, A^L^-B^L^, A^L^-B^L^}; {1, 1, 0, 0}, {0, 0, 1, 1}, {1, -1, 0, 0}, {0, 0, 1, -1}}.

Similarly, for inhibitory connections

$$\begin{array}{l} \overset{•}{W_{Z}}\approx g_{1}^{Z}W_{X}CW_{X}^{t}W_{Y}^{t}-g_{2}^{Z}W_{Z}W_{Y}W_{X}CW_{X}^{t}W_{Y}^{t}\\ g_{1}^{Z}\equiv \int_{-\infty }^{\infty }F^{Z}(s)\int D_{r}^{X}\int D_{u}^{Y}\int D_{r^{\prime }}^{X}h(r-s-u-r^{\prime }-d_{Z}-d_{Y})\\ g_{2}^{Z}\equiv \int_{-\infty }^{\infty }F^{Z}(s)\int D_{q}^{Z}\int D_{u}^{Y}\int D_{r}^{X}\int D_{u^{\prime }}^{Y}\int D_{r^{\prime }}^{X}h(r+u+q-s-u^{\prime }-r^{\prime }). \end{array}$$ (36)

We approximated with only two terms because the third term is negligible (S2D iv–vi Fig). If we assume $W_{Y}=\left(\begin{array}{cc} w_{d}^{Y} & w_{r}^{Y}\\ w_{r}^{Y} & w_{d}^{Y} \end{array}\right),$ and *g*_*2*_^*Z*^ = 0, then the synaptic weight change follows $\Delta w_{11}^{Z}-\Delta w_{12}^{Z}=\Delta w_{22}^{Z}-\Delta w_{21}^{Z}=c_{S}g_{1}^{Z}{\left(w_{s}^{X}-w_{s}^{X}\right)}^{2}\left(w_{d}^{Y}-w_{r}^{Y}\right)$. This means that if *g*_*1*_^*Z*^ is positive, reciprocal connections are enhanced (or inhibitory connections to the neurons coding a similar feature are enhanced), whereas for negative *g*_*1*_^*I*^, inhibitory connections develop non-reciprocally (i.e., lateral connections function as mutual inhibition between output excitatory neuron groups).

We have restricted our consideration to Hebbian STDP, but the properties of STDP on E-to-I and I-to-E connections are still debatable [58,59]. Although it is difficult to study all combinations of STDPs, we still provide analytical insights by investigating the behaviors of *g*_*1*_^*Y*^ and *g*_*1*_^*Z*^. S2E Fig shows the behaviors of four different types of STDP. This indicates that the anti-correlation detector type of E-to-I STDP [53] tends to cause non-feature-selective lateral connections. In addition, under the anti-coincidence detector type of I-to-E STDP [55], mutual inhibition structures would be preferred; however, the implication of our analytical method is limited, and further study will be necessary to fully understand the functions of the various types of STDP.

### Evaluation of the performance

#### Cross-correlation

We evaluated the performance by measuring the mean cross-correlation between the external sources and population activity of the output neurons. For time bin *Δt* = 10 ms, the activity of source *μ* is defined as $s_{\mu }^{k}=\frac{1}{\Delta t}\int_{k\Delta t}^{(k+1)\Delta t}s_{\mu }(t)dt$, and, similarly, the population activity of the output neuron group *ν* is $y_{\nu }^{k}(\tau_{D})=\sum_{j\in \Omega_{{}_{\nu }}^{Y}}\frac{1}{\Delta t}\int_{k\Delta t}^{(k+1)\Delta t}y_{j}(t+\tau_{D})dt$, where Ω_ν_^Y^ is a set of output neurons coding a source *ν*. For these, cross-correlation is defined as

$$c_{\mu \nu }(\tau_{D})\equiv \frac{1}{\sigma_{\mu }^{s}\sigma_{\nu }^{y}}\sum_{k=1}^{T_{c}/\Delta t}(s_{\mu }^{k}-{\bar{s}}_{\mu })(y_{\nu }^{k}-{\bar{y}}_{\nu }),$$

where ${\bar{s}}_{\mu }\equiv \frac{1}{T_{c}}\int_{T_{o}}^{T_{o}+T_{c}}s_{\mu }(t)dt$, ${\bar{y}}_{\nu }\equiv \frac{1}{T_{c}}\int_{T_{o}}^{T_{o}+T_{c}}y_{\nu }(t)dt$,$\sigma_{\mu }^{s}\equiv \sqrt{\sum_{k=1}^{T_{c}/\Delta t}{\left(s_{\mu }^{k}-{\bar{s}}_{\mu }\right)}^{2}}$ , and $\sigma_{\nu }^{y}\equiv \sqrt{\sum_{k=1}^{T_{c}/\Delta t}{\left(y_{\nu }^{k}-{\bar{y}}_{\nu }\right)}^{2}}$. We used *T*_*c*_ = 10 ms for the analysis. Correspondence between sources and output groups are arbitrary, and so the learned correlation should be given as $c(\tau_{D})\equiv \underset{\psi }{\max}\frac{1}{p}\sum_{\mu =1}^{p}c_{\mu \psi (\mu )}(\tau_{D})$ for all the *p*! number of combinations with function *Ψ* between sources and output groups. For example, when *p* = 2,$c(\tau_{D})=\max\left\{\frac{1}{2}\left[c_{A1}(\tau_{D})+c_{B2}(\tau_{D})\right],\frac{1}{2}\left[c_{A2}(\tau_{D})+c_{B1}(\tau_{D})\right]\right\}$. Although, in reality, supervised or reinforcement learning is necessary to perform this readout, for simplicity we did not implement readout neurons explicitly. In Fig 2F, we plotted $\underset{\nu }{\max}c_{B\nu }(\tau_{D})$ for the minor source B.

For the models with randomly connected lateral inhibition and (e+i) STDP, we defined output neuron *j* as belonging to Ω_μ_^Y^ if

$$\frac{1}{\left|\Omega_{\mu }^{X}\right|}\sum_{i\in \Omega_{\mu }^{X}}w_{j\mu }^{X}>\alpha_{th}\underset{\nu \neq \mu }{\max}\left\{\frac{1}{\left|\Omega_{\nu }^{X}\right|}\sum_{i\in \Omega_{\nu }^{X}}w_{j\nu }^{X}\right\}$$

for α_th_ = 1.5, and the cross-correlation was calculated based on Ω_μ_^Y^.

#### Mutual information

Based on the discretized hidden external source/output neuron activity *s*_*μ*_^*k*^, *y*_*ν*_^*k*^, we defined the binary variables

$${\hat{s}}_{\mu }^{k}\equiv \{\begin{array}{l} 1\text{ (if }s_{\mu }^{k}\text{>}{\bar{s}}_{\mu }^{k}+\sigma_{\mu }^{s}\text{)}\\ 0\text{ (otherwise)} \end{array},\;{\hat{y}}_{\nu }^{k}\equiv \left\{ \begin{array}{l} 1{\text{ (if y}}_{\nu }^{k}\text{>}{\bar{y}}_{\nu }^{k}+\sigma_{\nu }^{y}\text{)}\\ 0\text{ (otherwise)} \end{array}\right.$$

Based on these variables, the states at time *k* can be defined as ${\hat{s}}^{k}\equiv ({\hat{s}}_{1}^{k},...,{\hat{s}}_{p}^{k})$,${\hat{y}}^{k}\equiv ({\hat{y}}_{1}^{k},...,{\hat{y}}_{p}^{k})$. Therefore, the probability that the external state takes one particular state is $p_{s}(\hat{s}={\hat{s}}^{\prime })\equiv \frac{1}{T_{c}/\Delta t}\sum_{k=1}^{T_{c}/\Delta t}{\left[{\hat{s}}^{k}={\hat{s}}^{\prime }\right]}_{tof}$, where [X]_*tof*_ takes 1 if X is true, otherwise it takes 0, for the statement X. Therefore, mutual information can be defined as

$$MI\equiv \sum_{{\hat{s}}^{\prime }}\sum_{{\hat{y}}^{\prime }}p_{sy}(\hat{s}={\hat{s}}^{\prime },\hat{y}={\hat{y}}^{\prime })\log_{2}\left(\frac{p_{sy}(\hat{s}={\hat{s}}^{\prime },\hat{y}={\hat{y}}^{\prime })}{p_{s}(\hat{s}={\hat{s}}^{\prime })p_{y}(\hat{y}={\hat{y}}^{\prime })}\right).$$

## Supporting Information

S1 Fig Simulations with the leaky integrate-and-fire model. (A) Synaptic weight developments at the feedforward connection. (B) Cross-correlation and mutual information calculated for various delays. Both values were calculated by averaging five independent simulation results. (C) Development of two values for the simulation shown in (A). (D) PSTH of the membrane potential calculated for gray areas in (A). (E) Peristimulus time histogram (PSTH) of the firing probability for the same simulation.
(EPS)

S2 Fig Spike-timing-dependent plasticity (STDP) at lateral connections shapes network structure. (A, B) Synaptic weight development when the number of external inputs is three (A) and four (B). Thick lines represent averages over all synapses, and thin lines represent individual synaptic weights. Colors represent detected sources for output neurons (left) and inhibitory neurons (middle right). (C) Relationship between the number of inhibitory neurons and the lateral structure. (D) Propagation of structure. i to iii correspond to lateral excitatory connections, and iv to vi correspond to feedback inhibitory connections. (E) Analytic results for various types of STDP.
(EPS)

S3 FigThe effects of noise in the model with exponential correlation kernel.(A) Cross-correlations among input neurons responding to the same source calculated from simulated data for three different correlation timescale parameters θ_t_. Note that in Fig 3, we used θ_t_ = 0.5, 2.0, 4.0 ms, while here we used θ_t_ = 1.0, 3.0, 5.0ms. (B, C) The correlation kernels *g*_*e1*_^*X*^, *g*_*e2*_^*X*^ (B) and their ratio *g*_*e1*_^*X*^/*g*_*e2*_^*X*^ (C) are shown for the kernels *g*_*e1*_^*X*^ and *g*_*e2*_^*X*^ that were calculated from Eq (30) with $\phi_{e}(t)=e^{-t/\theta_{t}}/\theta_{t}$. (D,E) The effects of random noise (D) and crosstalk noise (E) at various correlation timescales.(EPS)

S1 Auditory File Demonstration of blind source separation. 0’00”–0’43”: Independent auditory signals (10 s each); 0’44”–0’54”: Mixed auditory signal; 0’55”–1’38”: Decoded independent signals.
(MP3)
